# Wide Range of the Prevalence and Viral Loads of Porcine Circovirus Type 3 (PCV3) in Different Clinical Materials from 21 Polish Pig Farms

**DOI:** 10.3390/pathogens9050411

**Published:** 2020-05-25

**Authors:** Aleksandra Woźniak, Dagmara Miłek, Tomasz Stadejek

**Affiliations:** Department of Pathology and Veterinary Diagnostics, Institute of Veterinary Medicine, Warsaw University of Life Sciences—SGGW, Nowoursynowska 159C, 02-776 Warsaw, Poland; aleksandra_wozniak1@sggw.edu.pl (A.W.); dagmara_milek@sggw.edu.pl (D.M.)

**Keywords:** PCV3, viremia, shedding with feces, oral fluid, reproductive failure, quantitative real time PCR

## Abstract

Porcine circovirus type 3 (PCV3) was described in different clinical cases and healthy pigs. However, little is known about its circulation in pig farms. In order to assess PCV3 prevalence in 21 Polish farms, serum, feces, and oral fluid samples were examined by quantitative real-time PCR. In total, 1451 pairs of serum and feces from the same animals, as well as 327 samples of oral fluids were analyzed. The results showed that PCV3 is more commonly detected in oral fluids (37.3% positives) than in serum (9.7% positives) or feces (15.0% positives) samples. The viral loads detected in these materials ranged from 10^2.5^–10^7.2^ genome equivalent copies/mL. Although in most farms PCV3 was detected post weaning, in nine farms, the virus was also found in groups of suckling piglets, and in six of them viremia was detected. In four farms with reproductive failure, fetal materials were also obtained. PCV3 was detected in 36.0% of fetuses or stillborn piglets (9/25) with viral loads of 10^3.1^–10^10.4^ genome equivalent copies/mL. In summary, the virus circulation may show different patterns, and congenital or early infection is not uncommon. Precise quantification of PCV3 loads in clinical materials seems to be necessary for the study and diagnosis of the infection.

## 1. Introduction

Porcine circovirus type 3 (PCV3) belongs to the family *Circoviridae*, genus *Circovirus*. It is a small non-enveloped virus with a single-stranded DNA genome of 1999–2001 nucleotides in size [1]. Since its first detection in the USA [2,3], PCV3 has been found in many countries, suggesting its global distribution [4]. In order to elucidate the PCV3 role in swine health, multiple detection methods, mainly based on PCR, *in situ* methods, and direct or indirect ELISA tests have been developed and applied in field studies [4]. The virus was found in many types of diagnostic materials, such as different tissues, serum, and oral fluids collected from pigs with different health status. Porcine circovirus type 3 was detected in cases of cardiac and multisystemic inflammation [2]; porcine dermatitis and nephropathy syndrome (PDNS) [3,5,6]; respiratory disease [7,8,9,10]; congenital tremor in neonatal pigs [11]; periarteritis [6]; reproductive failure, such as abortion, stillbirths, and mummification of fetuses [6,12,13,14,15,16,17,18,19,20]; or gastrointestinal signs [9,10]. On the other hand, many reports described the detection of PCV3 in pigs without any specific clinical signs [9,21,22,23,24,25,26]. 

Despite the global distribution of PCV3 and hints of its role in contributing to different disease conditions, little is known about the dynamics of PCV3 infection in farm populations. The only two reports were from Poland, and these studies were focused on the overall virus detection in different age groups of pigs from several farms, and only serum pools were analyzed [21,22]. Such an approach is suitable for surveillance of the spread of any virus across farms, but in order to assess the impact of PCV3 on different pigs, the testing of individual samples is required. Surprisingly low PCV3 detection rates in individual serum samples from a few pools identified in our previous study encouraged us to perform further more detailed studies on PCV3 circulation in Polish pig herds [22].

The aim of this study was to assess the detection rates of PCV3 in serum, feces, and oral fluid samples collected from pigs of different ages, from 21 Polish farms. Additionally, an examination of the samples from stillborn piglets or aborted fetuses from four of those farms was performed. 

## 2. Results

### 2.1. Porcine Circovirus Type 3 Detection in Serum, Feces, and Oral Fluid Samples

**Figure 1:**
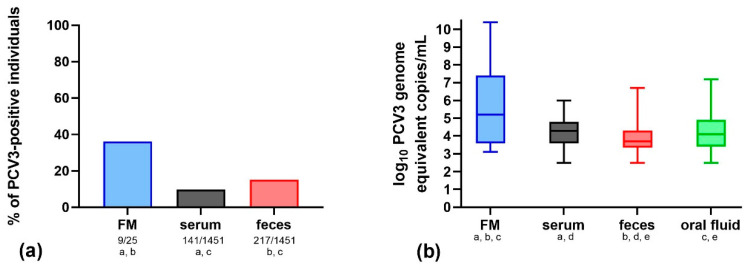
Detection of porcine circovirus type 3 (PCV3) (**a**) Percentage and proportion of PCV3-positive individuals (positive/all tested) based on quantitative real-time PCR (qPCR) of fetal, serum, and feces samples. Stillborn piglet or aborted fetus (FM - fetal material) was considered PCV3-positive if at least one sample reacted positively. Statistically significant differences (*p* < 0.05, Fisher’s exact test) are marked with subscripts under sample type (a–c). (**b**) Comparison of log_10_-transformed PCV3 viral loads (log_10_ genome equivalent copies/mL) in samples from FM, serum, feces, and oral fluids. The whisker plot shows the minimum and maximum. A statistical comparison was performed using the Mann–Whitney test. Statistically significant differences are marked with subscripts under sample type (a–e).

The results showed that the highest PCV3 detection frequency was observed in oral fluid samples (*p* < 0.05). In total, 122 out of 327 (37.3%) of the oral fluid samples were PCV3-positive. The PCV3 loads in the oral fluid samples ranged widely, from 10^2.5^ to as much as 10^7.2^ genome equivalent copies/mL, with a median of 10^4.1^ genome equivalent copies/mL (Figure 1, Table 1). 

The porcine circovirus type 3 detection rate in feces was significantly lower (*p* < 0.05), and the virus was found in 217 out of 1451 (15.0%) samples. Additionally, the log_10_ of PCV3 loads in feces (from 10^2.5^ to 10^6.7^; median = 10^3.7^ genome equivalent copies/mL) was significantly different (*p* < 0.05) than in oral fluid (Figure 1, Table 1).

Interestingly, serum was the sample type with significantly (*p* < 0.05) the lowest PCV3 detection rate. Overall, only 141 out of 1451 (9.7%) serum samples were positive for PCV3. The log_10_ of PCV3 loads in serum (from 10^2.5^ to 10^6.0^ genome equivalent copies/mL) were significantly different (*p* < 0.05) (median= 10^4.3^ genome equivalent copies/mL) than in feces (Table 1, Figure 1). The differences between the log_10_ of PCV3 loads in serum and oral fluid samples were not statistically significant (*p* > 0.05) (Figure 1).

### 2.2. Diverse Circulation Patterns of PCV3 in Different Farms

The sampled pig herds were found to exhibit very different patterns of PCV3 detection in serum, feces, and oral fluid samples. The PCV3 prevalence in oral fluids ranged from 12.5% (2 out of 16) in farm B to 100.0% in farm PA (Table 1). Porcine circovirus type 3 was most prevalent in fecal samples obtained from farm SU (50.0%, 30 out of 60). Interestingly, in farms KU, WA, WT, and RO, none of the examined fecal samples reacted positive for PCV3 (Table 1). The PCV3 detection rate in serum samples was the highest in farm AK (27.4%, 20 out of 73) and the lowest in farm BA, where only 1.3% (1 out of 80) of the serum samples were PCV3-positive (Table 1).

**Figure 2:**
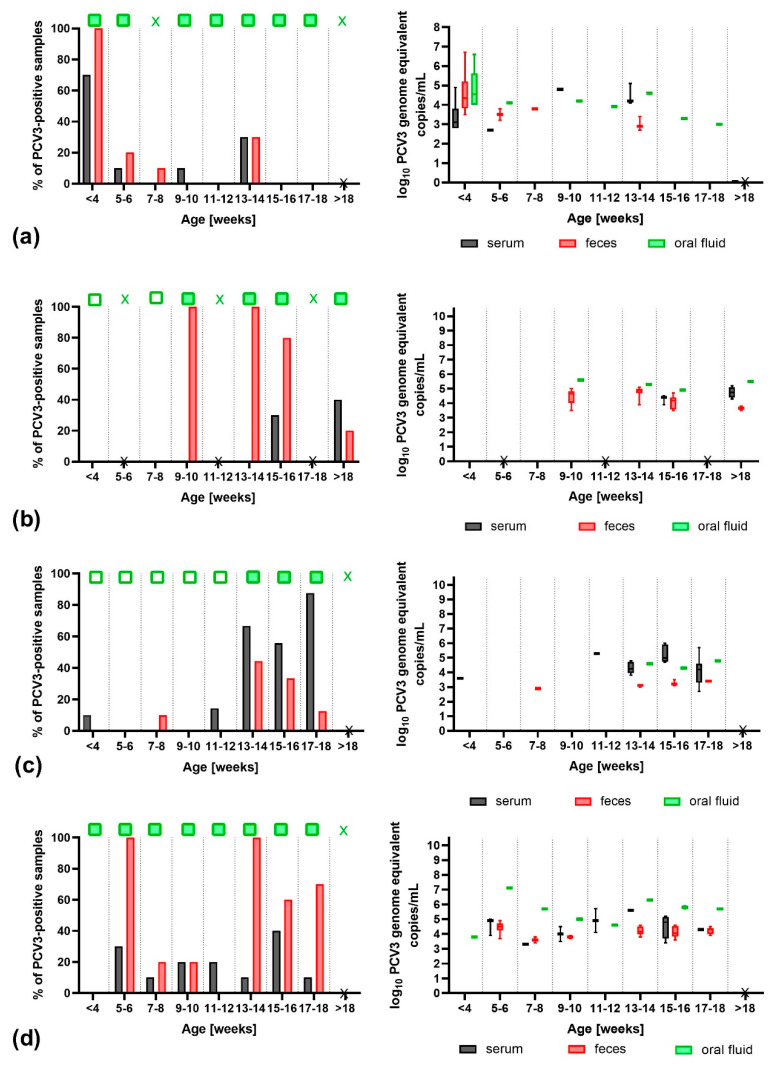
Percentages of porcine circovirus type 3 (PCV3)-positive samples and log_10_-transformed PCV3 viral loads (log_10_ genome equivalent copies/mL) in different age groups in farms PA (**a**), SU (**b**), AK (**c**), and PR (**d**). Age groups with at least one PCV3-positive oral fluid are marked with a solid green square. An empty green square indicates that oral fluids were negative for PCV3. The whisker plot shows the minimum and maximum. “X” on axis X indicates the age group, which was not sampled. A green mark “X” indicates a group where oral fluid was not obtained.

There were also important differences observed in the patterns of PCV3 circulation in different age groups. In farm PA, the PCV3 detection rate was the highest in 3-week-old piglets, where 70.0% (7 out of 10) of the serum samples and all fecal and oral fluid samples were positive (Figure 2a). On the other hand, in farms SU and AK, PCV3 in serum appeared in 15–16- and 11–12-week-old pigs, respectively (Figure 2b,c). In farm PR, the number of PCV3-viremic pigs was similar across all age groups (Figure 2d). The details of the PCV3 detection patterns in all the farms are presented in Figure S1 (Supplementary Materials).

### 2.3. Porcine Circovirus Type 3 in Fetal Material from Cases of Reproductive Failure

Porcine circovirus type 3 was detected in 9 out of 25 (36.0%; PCV3 viral loads 10^3.1^–10^10.4^ with median of 10^5.2^ genome equivalent copies/mL of sample) stillborn piglets or aborted fetuses, from four farms reporting reproductive failure (Figure 1, Table 2). Interestingly, in farm PR, all of the tested samples collected from four stillborn piglets were PCV3-positive, and PCV3 loads as high as 10^10.4^ genome equivalent copies/mL were detected. Porcine reproductive and respiratory syndrome virus (PRRSV) was not detected in any fetal sample (Table 2, Table S1). Porcine circovirus type 2 (PCV2) was found in two farms in six stillborn piglets or aborted fetuses (24.0%) and the viral load ranged from 10^3.8^–10^5.5^ genome equivalent copies/mL (median of 10^4.4^ genome equivalent copies/mL) (Table 2). In only 2 out of 25 (8.0%) stillborn piglets or aborted fetuses, both, PCV3 and PCV2, were detected (Table S1).

## 3. Discussion

Porcine circovirus type 3 is widely distributed in the world. Numerous studies described its detection in pigs with different health conditions, as well as in animals without any specific clinical signs [4]. The induction of clinical signs similar to PDNS after intranasal inoculation with PCV3 infectious clone was the first experimental evidence of the virus’ pathogenicity [5]. On the other hand, a higher PCV3 detection rate in pigs without any clinical signs than in unhealthy pigs was reported [25]. Information about PCV3 circulation in pigs of different ages from the same farms is limited to pooled serum samples [21,22]. While such studies provided a great deal of information about PCV3 circulation on a farm level, they did not reveal the virus’ spread among individual pigs. Thus, little information could be inferred regarding the pathogenic potential of the virus for individuals. In order to fill this gap in the knowledge, in the present study, different cross-sectionally obtained clinical samples from 21 conventional Polish pig farms were analyzed with quantitative real-time PCR (qPCR). Additionally, samples from stillborn piglets or aborted fetuses from four farms with cases of reproductive failure were examined.

The welfare and economic bonuses of testing oral fluids are well known. It was shown to be very useful for the detection and monitoring of PRRSV, influenza A virus (IAV), and PCV2 [27,28,29,30,31]. Porcine circovirus type 3 detection in oral fluids was previously described in Korea and China [23,26]. Kwon et al. (2017) reported that 44.2% (159 out of 360) of the tested oral fluids collected from weaned, growing, finisher, and sick pigs were PCV3-positive [23]. In China, PCV3 was detected only in 12.3% (39 out of 318) of the samples collected from sows and commercial pigs [26]. Unfortunately, the viral loads were not determined in those studies. In the present study, 37.3% of the oral fluids from 3–21-week-old pigs were positive for PCV3. Additionally, PCV3 viral loads were found to widely range from 10^2.5^ to as much as 10^7.2^ genome equivalent copies/mL (median = 10^4.1^ genome equivalent copies/mL) (Figure 1, Table 1). The detection rates and viral loads of PCV3 in oral fluids are much lower than in the case of PCV2, which was detected in 64.5% (10^4.3^–10^8.7^; median = 10^7.2^ genome equivalent copies/mL) and 46.1% (10^3.7^–10^7.4^; median = 10^5.5^ genome equivalent copies/mL) of samples, from farms non-vaccinated and vaccinated against PCV2, respectively [31]. 

The detection frequency of PCV3 in oral fluids was not reflected in the testing of serum (9.7%) and feces (15.0%). As these samples were obtained from the same individuals, the comparison of PCV3 viremia and shedding with feces was feasible. Interestingly, only 30.5% (43 out of 141 PCV3-positive serum samples) of viremic pigs shed PCV3 with feces. Inversely, only 19.8% (43 out of 217 PCV3-positive feces samples) of pigs shedding PCV3 with feces showed viremia. The discrepancy between the viremia and shedding with feces was previously described for PCV2 [31] and it may suggest that both, PCV2 and PCV3, can replicate, or persist, locally in the alimentary tract, causing little or no viremia. This discrepancy may also be explained by the very low virus levels in some of these materials, below the sensitivity of the employed test estimated at 10^2.4^ genome equivalent copies/mL of a sample, which could lead to the discordant results of the testing of the pairs of sera and feces from some individuals.

Several studies described PCV3 detection in different sample types from pigs at varied production phases [9,21,22,32]. Our previous studies showed that PCV3 prevalence was generally the highest in finishers (>9-week-old) [21,22]. However, when individual samples from different age groups were analyzed, more complex patterns of PCV3 circulation were detected. For example, in farm PA, very early PCV3 infection was observed, and 70.0% of 3-week-old piglets were already viremic, with the viral load ranging from 10^2.8^–10^4.9^ with a median of 10^3.1^ PCV3 genome equivalent copies/mL, while in some farms, PCV3 was not detected in the youngest animals (Figure S1). To sum up, on 6 out of 21 farms (28.6%), PCV3 viremia was found in at least one piglet (Figure S1). The PCV3 detection frequency in young piglets may reflect the infectious and immune status of their mothers, passing either PCV3 or protective levels of specific antibodies to their progeny. Kedkovid et al. (2018) showed that colostrum can also contain PCV3, which could lead to the infection of piglets [33]. 

Many studies showed concurrent PCV3 infections with PRRSV, PCV2, torque teno virus (TTV), or porcine parvoviruses (PPVs) [4]. In theory, this co-incidence could result in the exacerbation of clinical signs in coinfected animals. However, low or possibly undetected (below 10^2.4^ genome equivalent copies/mL) PCV3 viremia in the majority of the farms and samples makes it unlikely that the virus could contribute to any disease in those farms. Additionally, our earlier study indicated no impact of PCV3 on PCV2 viremia [22]. On the other hand, in farm AK, a 15-week-old fattener had relatively high viremia (10^6.0^ PCV3 genome equivalent copies/mL), so a negative impact of this virus should not be ultimately excluded. Thus, a better understanding of the interplay between PCV3 and other pathogens may be achieved through the analysis of a larger number of herds, from different countries, experiencing different disease problems and healthy ones. Additionally, the availability of PCV3 isolates will open new perspectives to study the pathogenic and immunopathogenic potential of PCV3 [20,34]. 

There are several reports on the PCV3 detection in stillborn or mummified piglets, suggesting that the virus may contribute to reproductive failure [6,12,13,14,15,16,17,18,19,20]. In Brazil, nearly 97.0% (270 out of 276) of mummified fetuses examined were PCV3-positive, while in only 12 of them (4.4%) PCV3 was the only pathogen detected [16]. On the other hand, in Korea, PCV3 alone was detected in 6 out of 14 (42.9%) aborted fetuses and 2 out of 8 (25.0%) suckling piglets from one farm [17]. Although in these reports, PCV3 was confirmed as the only pathogen detected in cases of reproductive problems, the viral loads were not examined, so the evaluation of the level of the infection was not possible. It is known that, for example, PCV2 is often detected in fetal materials and newborns at a very low level (e.g., 10^4.0^ PCV2 copies/500 ng of tissue) [35] and it is commonly accepted that such a finding has very little diagnostic value and certainly does not prove the role of PCV2 in the disease.

In our study, PCV3 was detected in fetal material from four farms reporting reproductive failure. These samples were negative for PRRSV, and PCV2 was detected in six fetuses or stillborn piglets. For the confirmation of PCV2 reproductive disease (PCV2-RD), >10^7.0^/500 ng DNA of tissues from mummified or stillborn piglets is considered diagnostically significant [36]. It is difficult to directly relate the levels of PCV2 detected in the present study (10^3.8^–10^5.5^; median = 10^4.4^ PCV2 genome equivalent copies/mL) to the aforementioned reference, and to exclude or confirm the role of this virus in the observed reproductive failure. However, very high PCV3 loads in some samples, up to 10^10.4^ genome equivalent copies/mL in farm PR, strongly suggest the role of PCV3 in the condition. So, our results support the previous findings, where PCV3 loads, as high as 10^9.0^ to 10^11.0^ copies/g of tissues [12] or trillions of genome equivalent copies/mL of tissue homogenate [6], were found in cases of reproductive failure. The high detectability and viral loads of PCV3 in fetal material have to be considered a strong indication of intrauterine infections leading to reproductive disease. 

Understanding PCV3’s role for swine health remains a challenge. Our studies performed in Polish farms showed that despite the wide spread of the virus between the farms, the infection occurs in relatively few weaners and fatteners, and the level of viremia is generally low. As such, together with the fact that this virus does not seem to impact the course of the PCV2 infection [22], it can be concluded that the significance of PCV3 for growing pigs is minimal. However, it has to be stressed that this conclusion is based on the results of the analysis of relatively few farms from a single country, and has to be considered preliminary. On the other hand, our results provided more important evidence that PCV3 should be seriously considered as a reproductive pathogen. Highly diverse profiles of PCV3 detection pre- and post-weaning suggest differences between sow herds regarding the PCV3 infection status and immunity. It is plausible that in most of the analyzed farms, the maternal immunity against PCV3-protected pigs against the infection until the growing or fattening period. However, the detection of PCV3 in fetal materials and suckling piglets in some farms suggested the opposite. More studies are needed to assess whether this problem can be considered serious, and whether there is a need for a vaccine to control it. In summary, quantification of PCV3 viral loads in clinical materials seems to be necessary for the study and diagnosis of PCV3 infection, in order to validate the role of the virus in inducing health problems, similarly to the criteria established for PCV2. 

## 4. Materials and Methods

### 4.1. Samples

The study was performed on archival samples from 21 commercial Polish pig farms that previously were found to be positive for PCV3. The farms varied in size, production type, general hygiene level, and health status. Serum, feces, and oral fluid samples were obtained from random pigs, mainly of about 3–21 weeks of age, as a part of a routine diagnostic or monitoring program; thus, the approval of the local ethic committee was not required (Figure S1). From each age group, 6 to 10 blood and fecal samples were obtained. Fecal samples were collected by rectal swabbing at the same time as bleeding. Additionally, one oral fluid sample was obtained from each pen of pigs where blood and feces were collected, using a standard cotton rope method [37]. As this collection method is ineffective in suckling piglets, oral swabs were obtained from this group. Additionally, sections of different internal organs (heart, lungs, kidney, liver, spleen), body cavity fluid, or umbilical cords from 25 fetuses or stillborn piglets from four farms were collected. 

Serum, feces, and oral fluids were processed as previously described [31,37]. The materials from each fetus or a stillborn piglet were pooled, homogenized in phosphate-buffered saline (PBS), vortexed for five minutes, and clarified by a short centrifugation. From each fetus or stillborn piglet, 1–4 pooled samples were prepared consisting of different organs or body cavity fluid (Table S1). All the samples were stored in −20 °C until usage. 

In total, serum and feces from 1451 pigs, different materials from 25 stillborn piglets or aborted fetuses, as well as 327 oral fluids, from 21 farms, were available for testing for the presence of PCV3.

### 4.2. DNA Extraction and qPCR

DNA extraction and qPCR from serum, feces, and oral fluids from 3-week-old piglets was performed in two stages. First, equal volumes of the material were pooled by 4–6 (each pool corresponded to one pen of weaners or fatteners, or one litter of suckling piglets), 200 µL of which was extracted with a QIAmp DNA Mini Kit or QIAmp cador Pathogen Mini Kit (Qiagen, Hilden Germany) and tested by qPCR. Next, the samples from the pools that were PCV3-positive were extracted individually and tested with qPCR. Oral fluids from weaners and fatteners, and pooled fetal samples were also extracted and tested with the same methods.

Extracted DNA (2.0 µL) was amplified with the qPCR assay targeting ORF2 of PCV3, as previously described [3]. Quantification of the viral copies in the samples was performed using titrated plasmid containing ORF2 of PCV3 [22]. Viral loads inferred from the qPCR results were expressed as viral genome equivalent copies per milliliter of serum, fecal, oral fluid sample, or tissue homogenate (genome equivalent copies/mL). Samples with a cycle threshold (Ct) >37.0 were considered as negative.

Fetal samples were additionally tested for the presence of PRRSV and PCV2 with real-time PCR as described previously [31,38]. 

### 4.3. Statistical Analysis

Statistical analysis was performed using GraphPad Prism 8 for Windows (GraphPad Software, San Diego, CA, USA, www.graphpad.com). The prevalence of PCV3 in different diagnostic materials was compared using Fischer’s exact test. Comparison of log_10_-transformed PCV3 viral loads (log_10_ genome equivalent copies/mL) was made using the nonparametric Mann–Whitney test. A two-tailed *p*-value <0.05 was set as the statistically significant level.

## 5. Conclusions

Porcine circovirus type 3 is widely spread across Polish pig farms and most often detected in oral fluids. Although in most farms, PCV3 was detected with a low prevalence and only post weaning, suggesting the protective role of maternal immunity, the detection of viremia in suckling piglets from six farms shows that the infectious and immune status can differ between sow herds. The detection of PCV3 in fetuses or stillborn piglets, sometimes with high loads, is more proof of the above. In summary, our results indicated that there is a need for similar studies to be performed in different countries, in herds of different clinical situations. Importantly, they should involve precise quantification of PCV3 loads in clinical materials, in order to validate the hypotheses on the pathogenic role of this virus, similar to that established for PCV2.

## Figures and Tables

**Table 1:** Farm characteristics and summary results of quantitative real-time PCR (qPCR) for porcine circovirus type 3 (PCV3) in serum, feces, and oral fluids. Viral loads were log_10_-transformed. Samples with Ct > 37.0 were considered negative. The farms reporting reproductive problems (stillbirths and abortion), where fetal samples were obtained, are marked with an asterisk.

Farm ID	Herd Size	Vaccination Against PCV2	% of PCV3-Positive Samples (Positive/All Tested)		
			PCV3 Viral Load: (Minimum-Maximum; Median) [log_10_ Genome Equivalent Copies/mL]		
			Serum	Feces	Oral Fluid
KR	8200	Piglets	3.8, (3/80)	6.3 (5/80)	33.3 (6/18)
			4.1–4.6; 4.3	2.8–3.1; 3.0	3.0–5.1; 3.4
AK	20-30	None	27.4 (20/73)	12.3 (9/73)	15.8 (3/19)
			2.7–6.0; 4.5	2.9–3.5; 3.2	4.3–4.8; 4.6
KU	65	Sows, piglets	13.3 (8/60)	0.0 (0/60)	13.3 (2/15)
			3.2–4.9; 4.4		2.8–6.4; 4.6
BY	5000	Piglets	11.3 (9/80)	12.5 (10/80)	43.8 (7/16)
			3.1–5.9; 3.8	3.2–5.0; 3.6	3.0–4.7; 4.2
SU	100	Sows, piglets	11.7 (7/60)	50.0 (30/60)	28.6 (4/14)
			3.9–5.2; 4.5	3.5–5.1; 4.7	4.9–5.6; 5.4
MI	500	Sows, piglets	15.5 (9/58)	12.1 (7/58)	30.8 (4/13)
			3.0–5.9; 3.5	2.9–4.1; 3.3	3.1–5.7; 3.6
KS*	180	Piglets	26.7 (16/60)	16.7 (10/60)	85.7 (6/7)
			3.2–5.5; 4.6	2.7–3.5; 3.4	3.5–5.6; 4.9
C	220	Piglets	12.0 (6/50)	10.0 (5/50)	33.3 (2/6)
			3.2–5.2; 4.3	3.7–4.7; 4.1	2.5–5.7; 4.1
PA*	1000	Piglets	15.0 (12/80)	20.0 (16/80)	100 (16/16)
			2.7–5.1; 3.7	2.7–6.7; 3.9	3.0–6.6; 4.2
PB	2300	Sows, piglets	11.4 (8/70)	37.1 (26/70)	88.2 (15/17)
			2.8–5.1; 4.5	2.7–4.6; 3.6	2.9–5.3; 3.5
GN	2250	Sows, piglets	4.0 (2/50)	14.0 (7/50)	41.2 (7/17)
			4.5–5.0; 4.8	3.1–4.9; 4.0	3.6–6.1; 4.4
DO	1800	Sows, piglets	11.3 (9/80)	12.5 (10/80)	41.2 (7/17)
			3.3–5.0; 3.7	3.1–4.2; 3.4	3.1–4.9; 3.4
ZA*	4950	Piglets	3.8 (3/80)	27.5 (22/80)	41.2 (7/17)
			3.0–3.4; 3.4	3.2–4.6; 3.7	3.6–5.2; 4.7
BA	600	Piglets	1.3 (1/80)	6.3 (5/80)	16.7 (3/18)
			4.3	2.6–3.9; 3.5	3.5–4.7; 4.0
B	220	None	2.9 (2/70)	7.1 (5/70)	12.5 (2/16)
			4.5–4.9; 4.7	3.4–4.0; 3.7	3.6–4.3; 4.0
PR*	390	Sows, piglets	17.5 (14/80)	46.3 (37/80)	50.0 (9/18)
			3.3–5.7; 4.6	3.4–4.9; 4.2	3.8–7.2; 5.7
WA	200	None	4.0 (2/50)	0.0 (0/50)	21.4 (3/14)
			3.5–4.8; 4.2		2.8–3.6; 3.0
WT	650	Sows, piglets	3.3 (2/60)	0.0 (0/60)	31.6 (6/19)
			3.4–5.5; 4.5		2.6–3.6; 3.1
AG	2400	Piglets	5.0 (4/80)	11.3 (9/80)	23.5 (4/17)
			2.5–4.0; 3.5	2.5–3.8; 2.8	3.2–5.3; 4.2
RO	800	Piglets	1.4 (1/70)	0.0 (0/70)	18.8 (3/16)
			4.4		3.4–4.0; 3.7
GR	3800	Piglets	3.8 (3/80)	5.0 (4/80)	35.3 (6/17)
			3.4–5.1; 4.2	2.8–4.4; 3.5	3.4–4.4; 4.0
**TOTAL**			**9.7 (141/1451)**	**15.0 (217/1451)**	**37.3 (122/327)**
			**2.5–6.0; 4.3**	**2.5–6.7; 3.7**	**2.5–7.2; 4.1**

**Table 2:** Summary of the results of quantitative real-time PCR (qPCR) for porcine circovirus type 3 (PCV3), porcine circovirus type 2 (PCV2), and porcine reproductive and respiratory syndrome virus (PRRSV) in pooled samples collected from stillborn piglets or aborted fetuses. Stillborn piglet or aborted fetus was considered PCV3-positive if at least one sample was positive for PCV3, PCV2, or PRRSV. Viral loads were log_10_-transformed. Samples with Ct>37.0 were considered negative.

Farm ID	PCV3		PCV2		PRRSV
	% of Positive Stillborn Piglets or Aborted Fetuses (Positive/All Tested)	Viral Loads in Fetal Samples (Minimum-Maximum; Median) [log_10_ Genome Equivalent Copies/mL]	% of Positive Stillborn Piglets or Aborted Fetuses (Positive/All Tested)	Viral Loads in Fetal Samples (Minimum-Maximum; Median) [log_10_ Genome Equivalent Copies/mL]	% of Positive Stillborn Piglets or Aborted Fetuses (Positive/All Tested)
KS	23.1 (3/13)	3.5–3.8, 3.6	38.5 (5/13)	3.8–5.5; 4.6	0.0 (0/13)
PR	100.0 (4/4)	4.6–10.4; 6.5	25.0 (1/4)	4.0	0.0 (0/4)
PA	50.0 (1/2)	3.6	0.0 (0/2)	-	0.0 (0/2)
ZA	16.7 (1/6)	3.1	0.0 (0/6)	-	0.0 (0/6)
**TOTAL**	**36.0 (9/25)**	**3.1–10.4; 5.2**	**24.0 (6/25)**	**3.8–5.5; 4.4**	**0.0 (0/25)**

## Supplementary Materials

The following are available online at https://www.mdpi.com/2076-0817/9/5/411/s1, Figure S1: Percentages of porcine circovirus type 3 (PCV3)-positive samples and log_10_-transformed PCV3 viral loads (log_10_ genome equivalent copies/mL) in different age groups from each farm. Table S1: Summary results of real-time PCR for porcine circovirus type 3 (PCV3), porcine circovirus type 2 (PCV2) and porcine reproductive and respiratory syndrome virus (PRRSV) in samples collected from stillborn piglets or aborted fetuses.
