# Supplementary material for: Wide Range of the Prevalence and Viral Loads of Porcine Circovirus Type 3 (PCV3) in Different Clinical Materials from 21 Polish Pig Farms
Source: Pathogens. 2020 May 25;9(5):411. doi: 10.3390/pathogens9050411 (PMC7281387; doi:10.3390/pathogens9050411)
Supplement: Supplementary file 1 [file pathogens-09-00411-s001.zip › supplementary figureS1_rev1_FINAL.pdf]

FARM KR

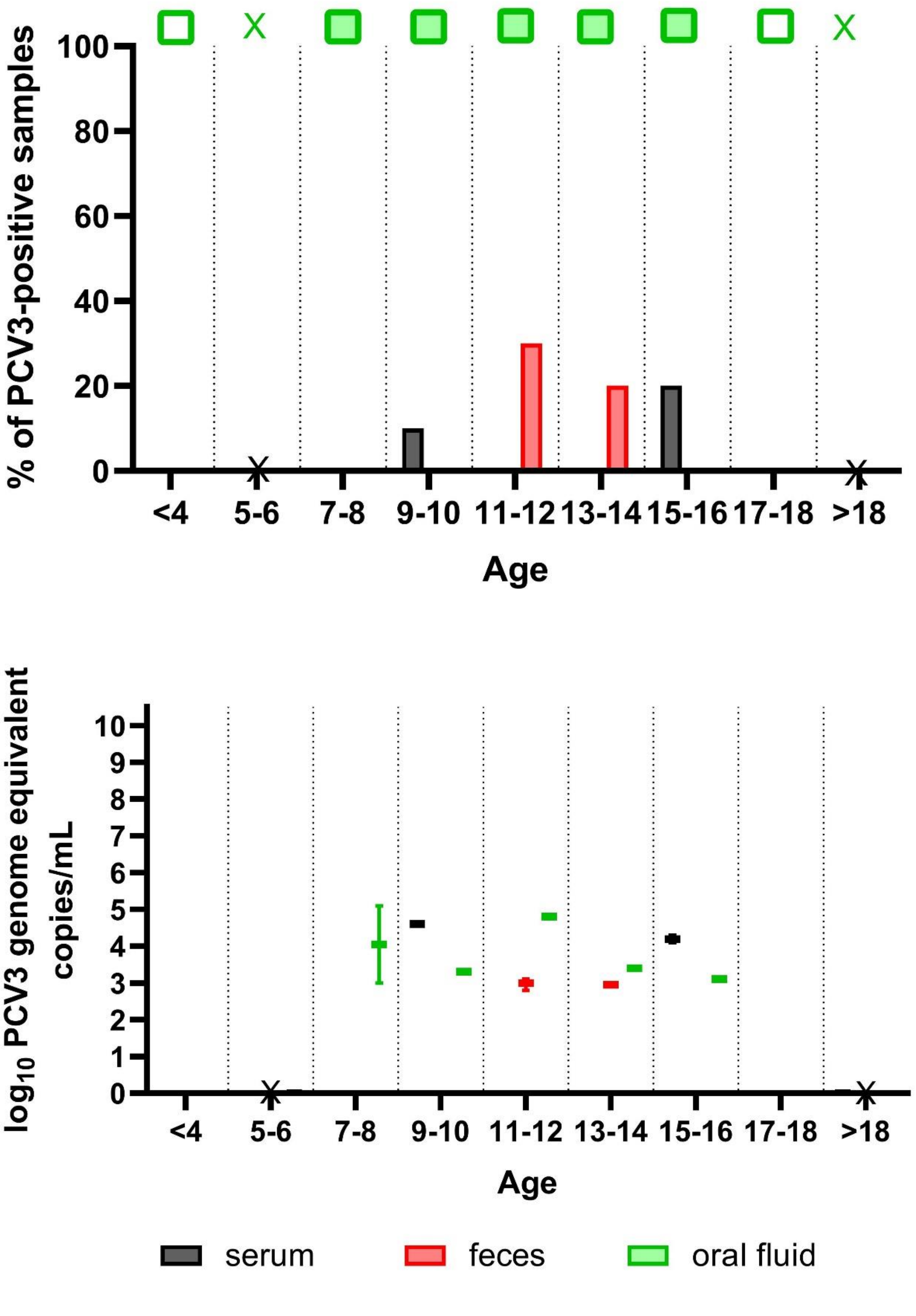

Figure S1: Percentages of porcine circovirus type 3 (PCV3)-positive samples and PCV3 viral loads (log_{10} genome equivalent copies/mL) in different age groups in examined farms. Age groups with at least one oral fluid PCV3-positive are marked with a solid green square. An empty green square indicates that oral fluids reacted negative for PCV3. The whisker plot shows minimum and maximum. "X" on axis X indicates age group, which was not sampled. A green mark "X" indicates a group, where oral fluid was not obtained. Age is expressed in weeks.

FARM AK

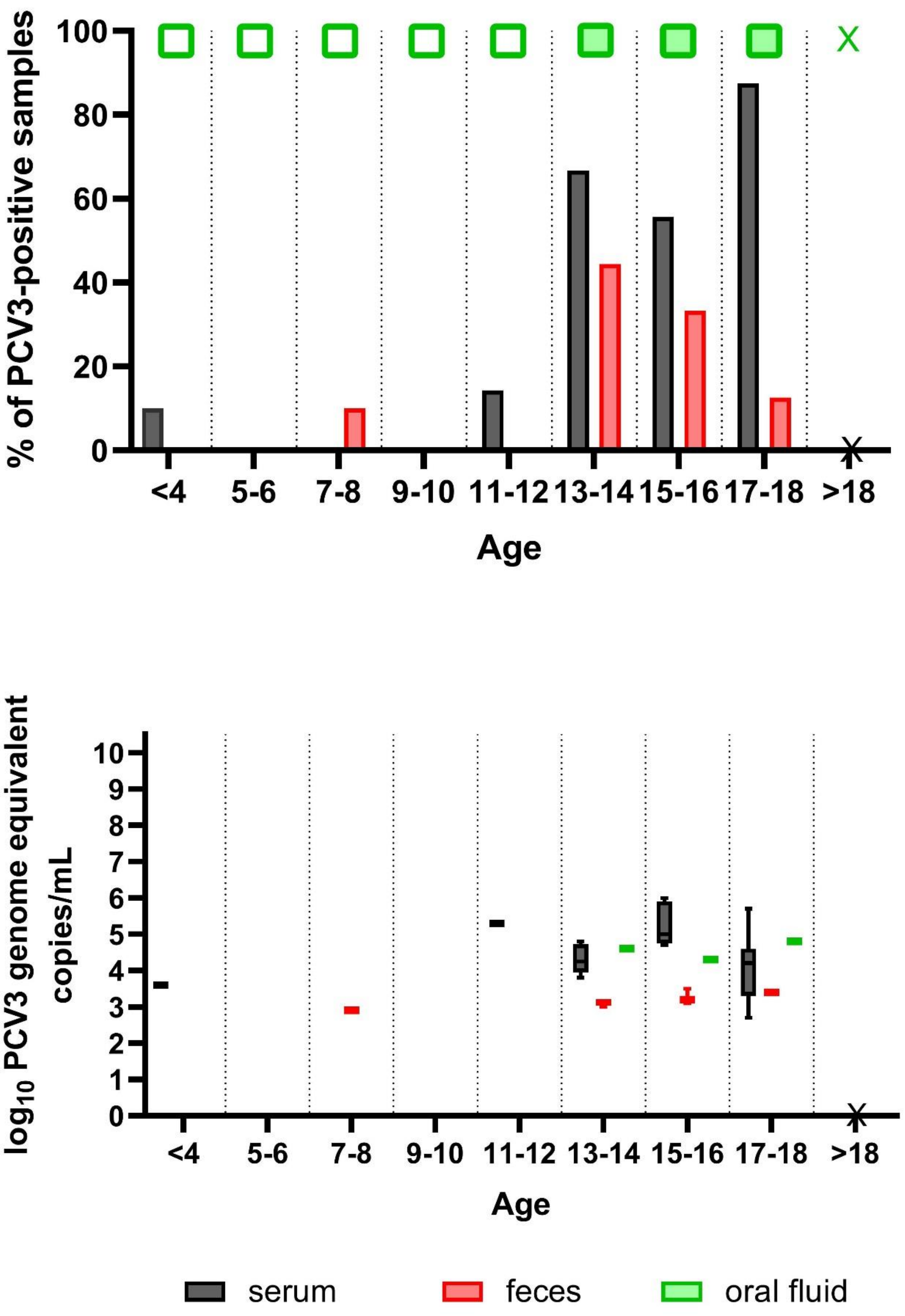

Figure S1: Percentages of porcine circovirus type 3 (PCV3)-positive samples and PCV3 viral loads (log_{10} genome equivalent copies/mL) in different age groups in examined farms. Age groups with at least one oral fluid PCV3-positive are marked with a solid green square. An empty green square indicates that oral fluids reacted negative for PCV3. The whisker plot shows minimum and maximum. "X" on axis X indicates age group, which was not sampled. A green mark "X" indicates a group, where oral fluid was not obtained. Age is expressed in weeks.

FARM KU

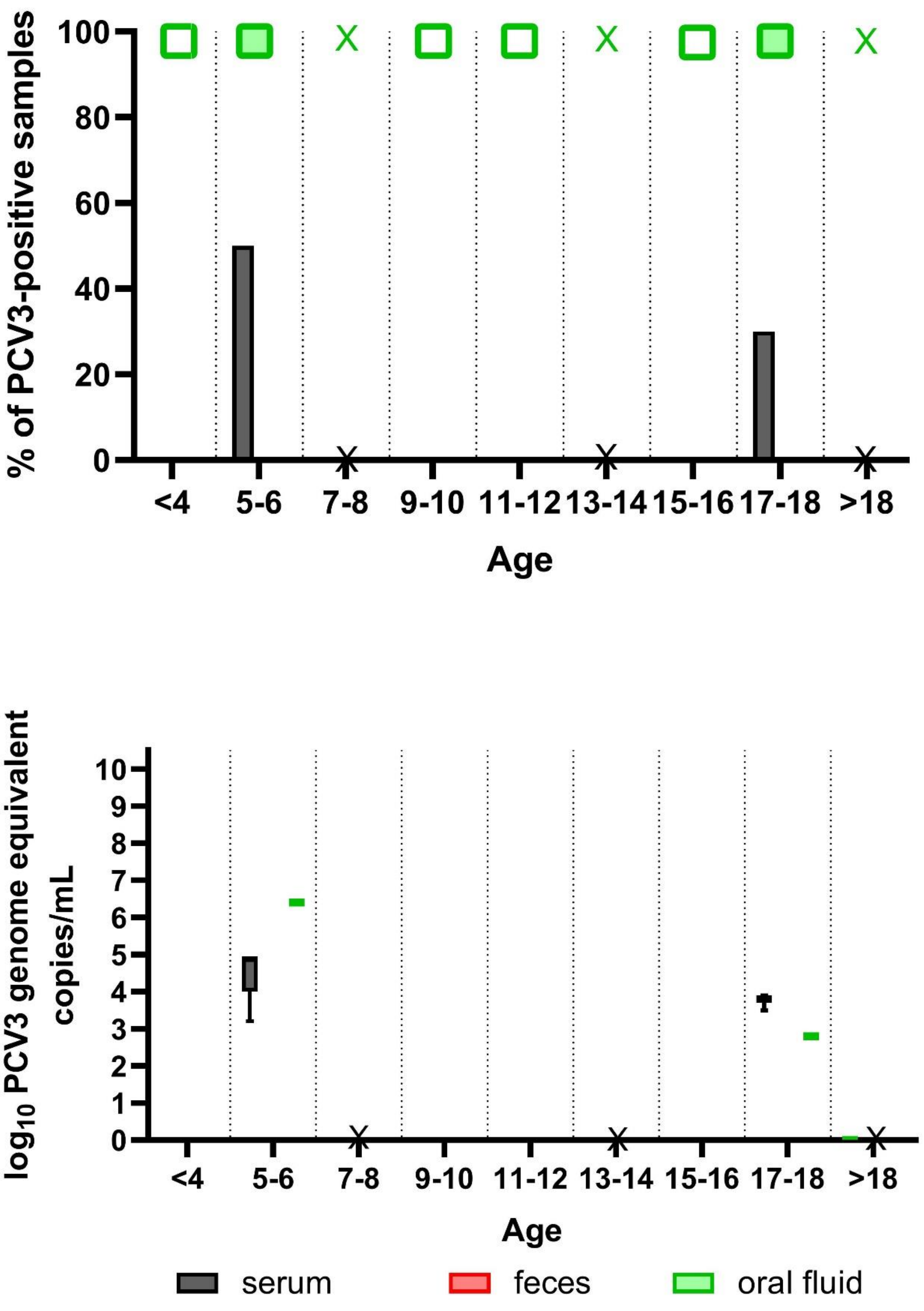

Figure S1: Percentages of porcine circovirus type 3 (PCV3)-positive samples and PCV3 viral loads ($\log_{10}$ genome equivalent copies/mL) in different age groups in examined farms. Age groups with at least one oral fluid PCV3-positive are marked with a solid green square. An empty green square indicates that oral fluids reacted negative for PCV3. The whisker plot shows minimum and maximum. "X" on axis X indicates age group, which was not sampled. A green mark "X" indicates a group, where oral fluid was not obtained. Age is expressed in weeks.

FARM BY

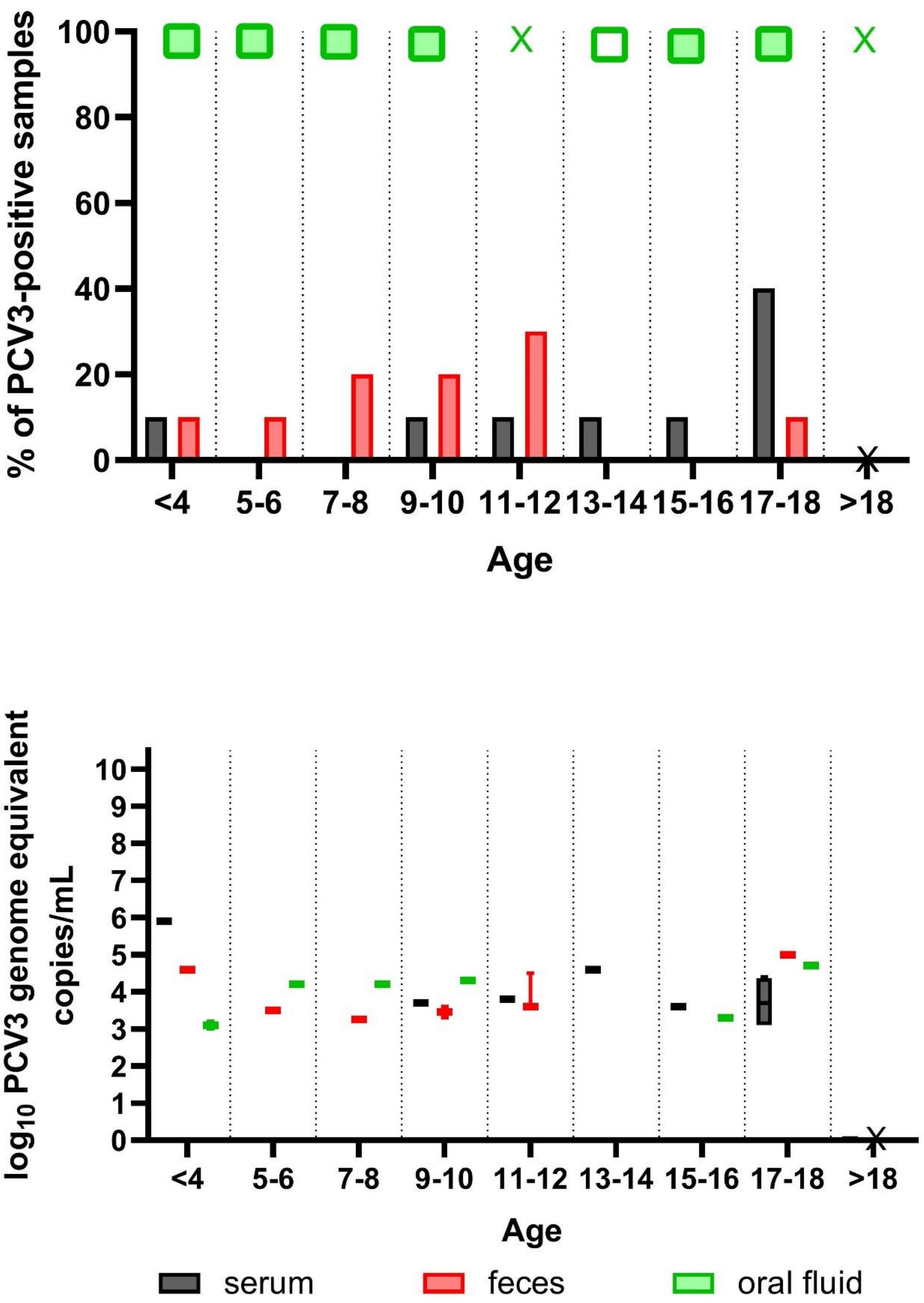

Figure S1: Percentages of porcine circovirus type 3 (PCV3)-positive samples and PCV3 viral loads (log_{10} genome equivalent copies/mL) in different age groups in examined farms. Age groups with at least one oral fluid PCV3-positive are marked with a solid green square. An empty green square indicates that oral fluids reacted negative for PCV3. The whisker plot shows minimum and maximum. "X" on axis X indicates age group, which was not sampled. A green mark "X" indicates a group, where oral fluid was not obtained. Age is expressed in weeks.

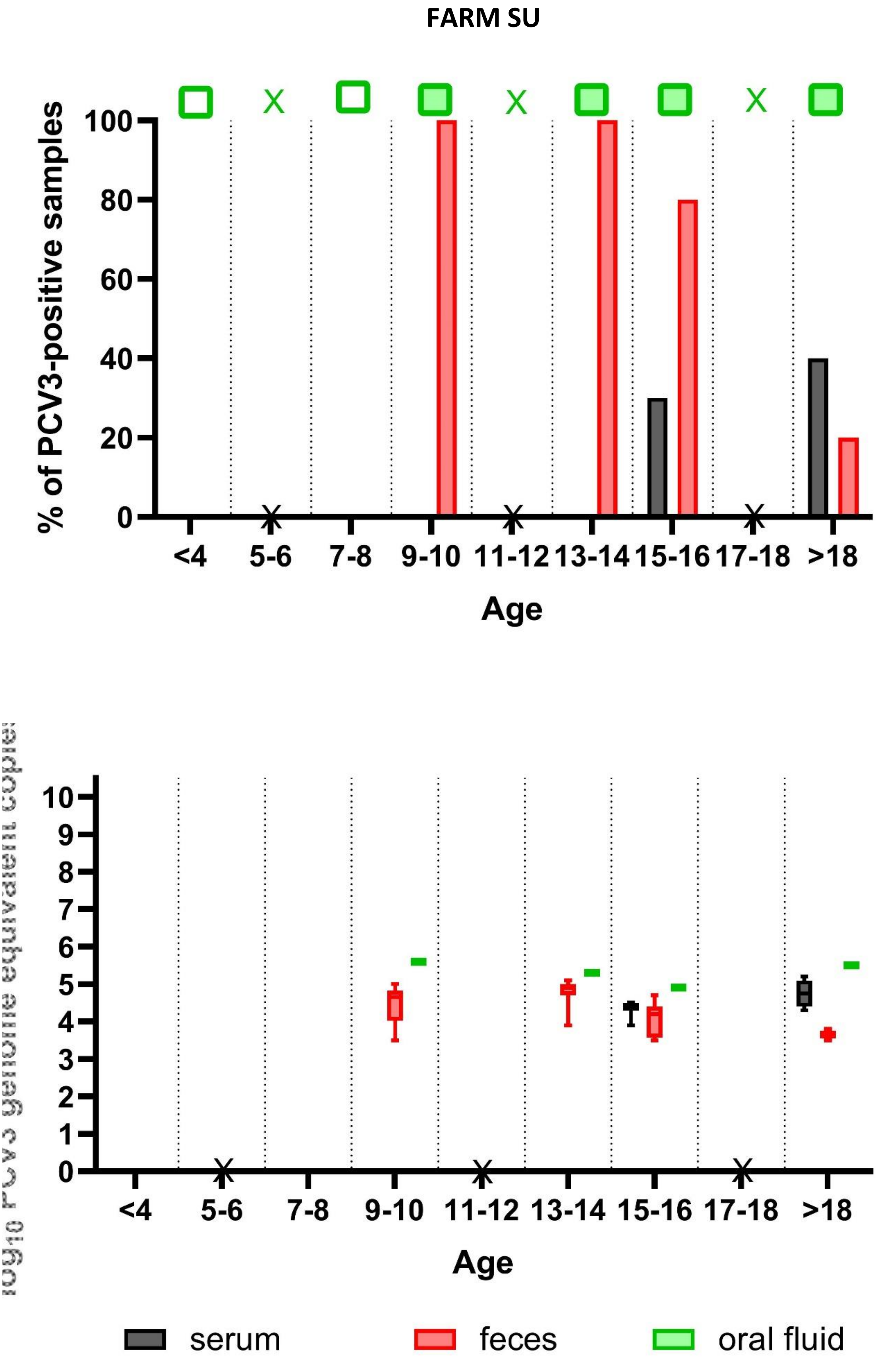

Figure S1: Percentages of porcine circovirus type 3 (PCV3)-positive samples and PCV3 viral loads (log_{10} genome equivalent copies/mL) in different age groups in examined farms. Age groups with at least one oral fluid PCV3-positive are marked with a solid green square. An empty green square indicates that oral fluids reacted negative for PCV3. The whisker plot shows minimum and maximum. "X" on axis X indicates age group, which was not sampled. A green mark "X" indicates a group, where oral fluid was not obtained. Age is expressed in weeks.

FARM MI

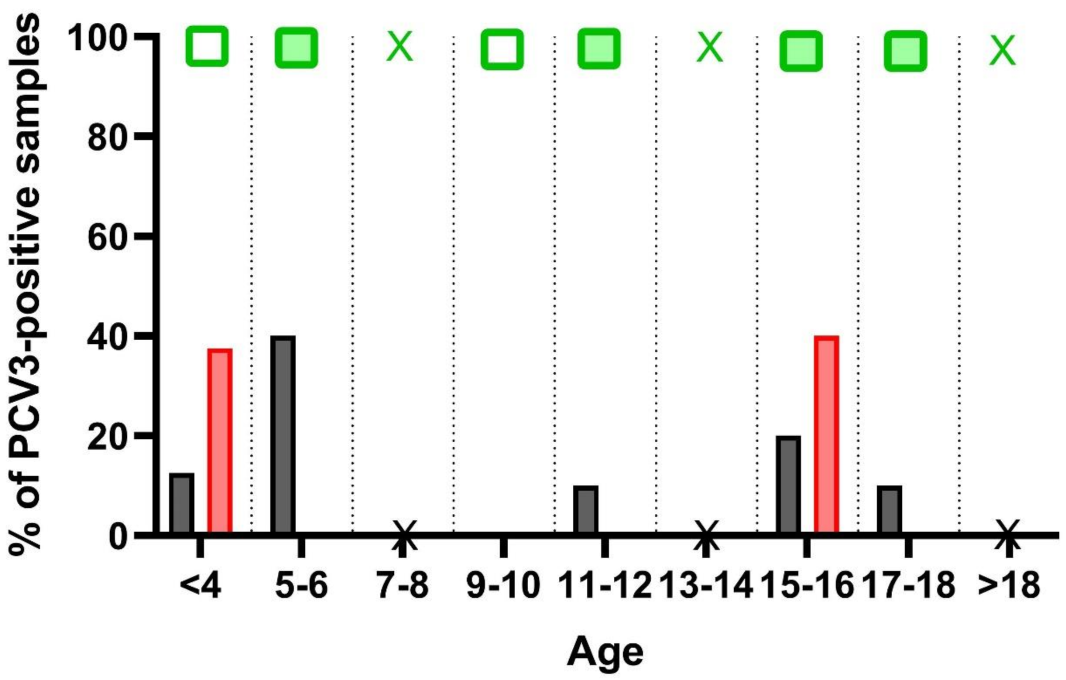

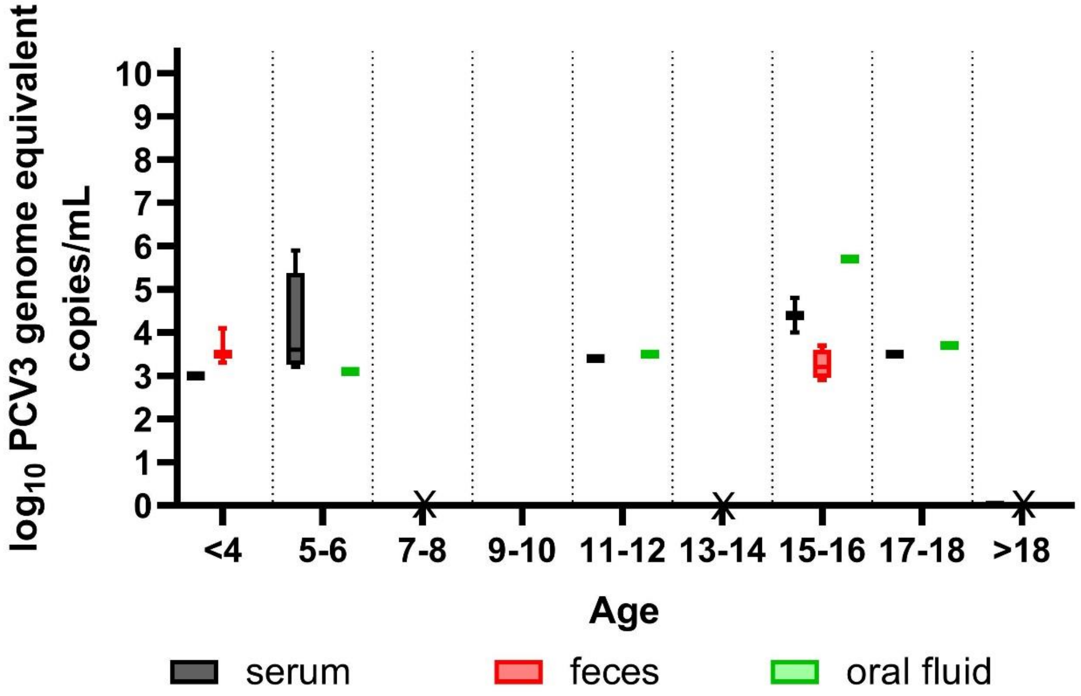

Figure S1: Percentages of porcine circovirus type 3 (PCV3)-positive samples and PCV3 viral loads ($\log_{10}$ genome equivalent copies/mL) in different age groups in examined farms. Age groups with at least one oral fluid PCV3-positive are marked with a solid green square. An empty green square indicates that oral fluids reacted negative for PCV3. The whisker plot shows minimum and maximum. "X" on axis X indicates age group, which was not sampled. A green mark "X" indicates a group, where oral fluid was not obtained. Age is expressed in weeks.

FARM KS

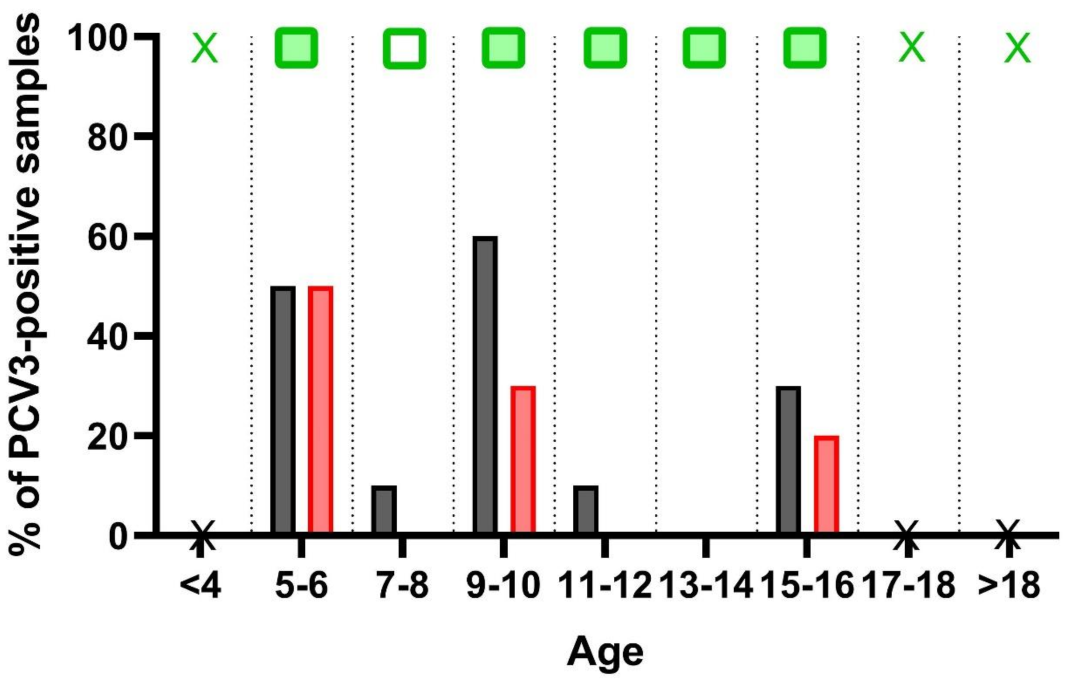

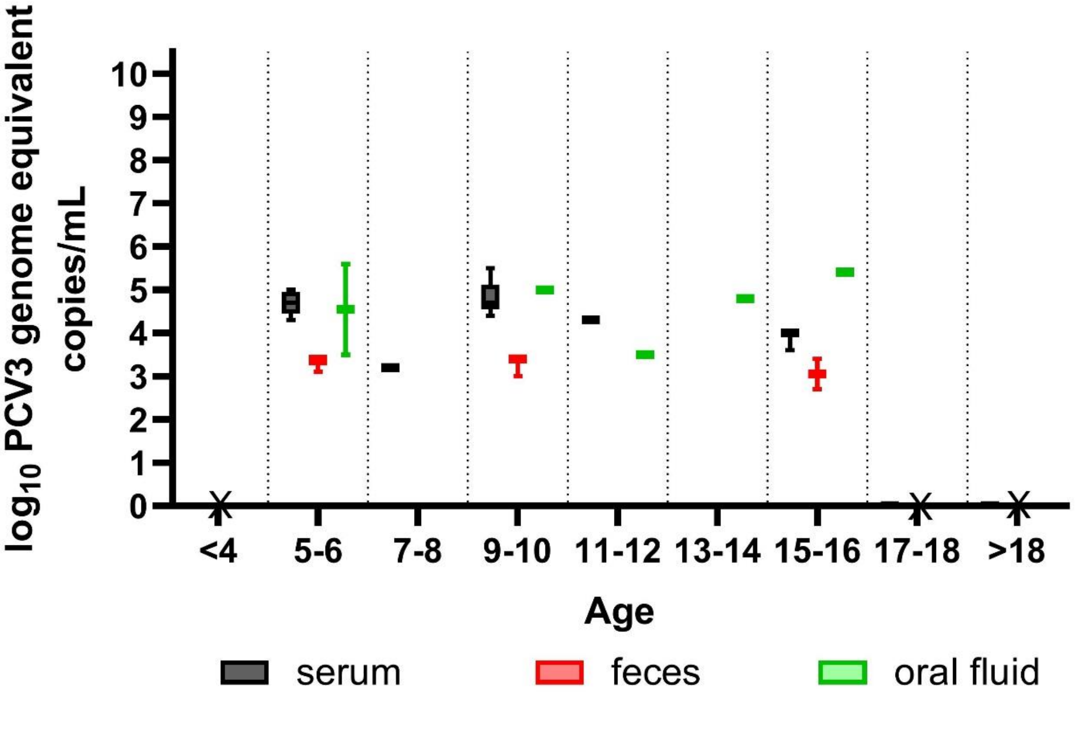

Figure S1: Percentages of porcine circovirus type 3 (PCV3)-positive samples and PCV3 viral loads (log_{10} genome equivalent copies/mL) in different age groups in examined farms. Age groups with at least one oral fluid PCV3-positive are marked with a solid green square. An empty green square indicates that oral fluids reacted negative for PCV3. The whisker plot shows minimum and maximum. "X" on axis X indicates age group, which was not sampled. A green mark "X" indicates a group, where oral fluid was not obtained. Age is expressed in weeks.

FARM C

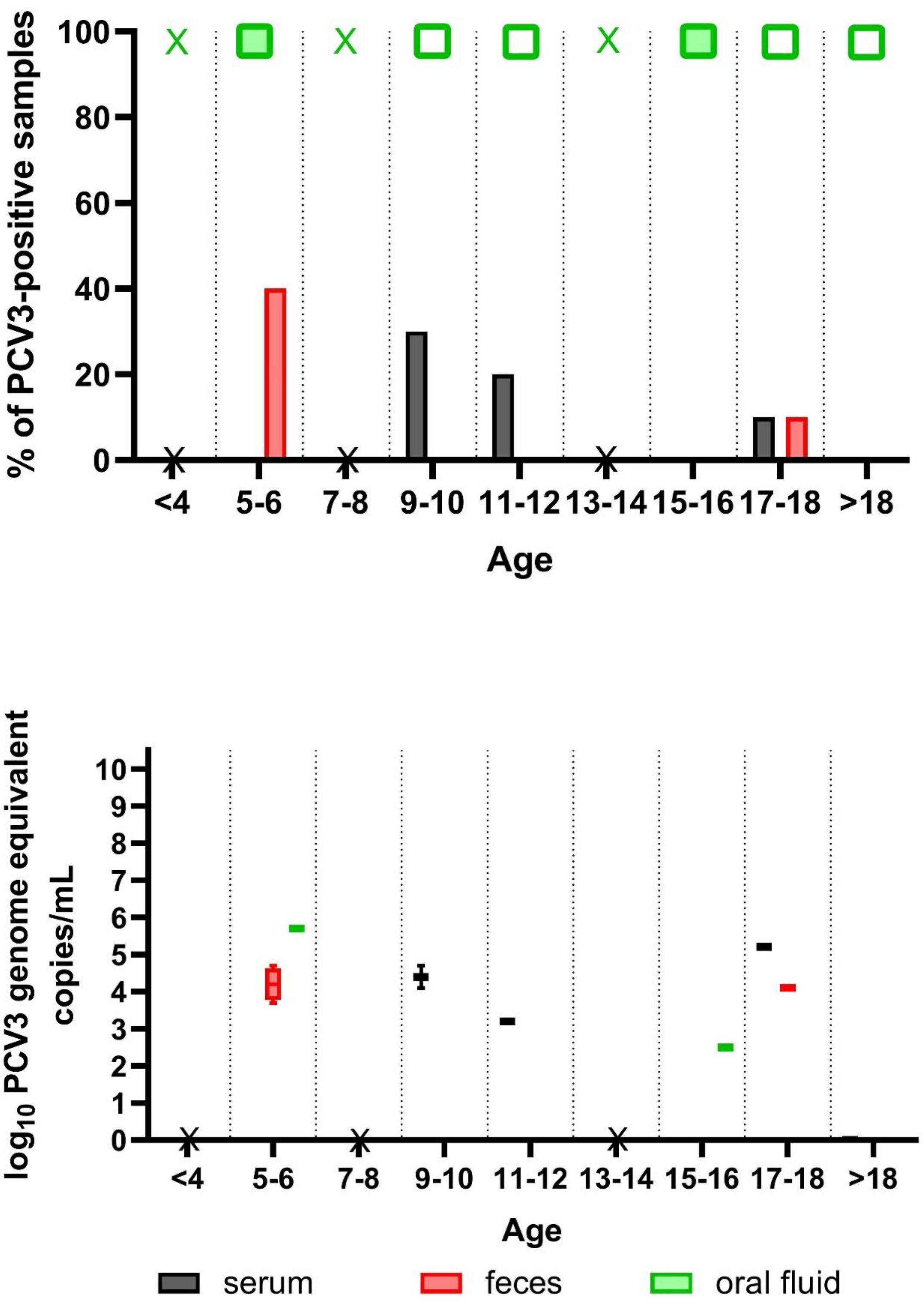

Figure S1: Percentages of porcine circovirus type 3 (PCV3)-positive samples and PCV3 viral loads (log_{10} genome equivalent copies/mL) in different age groups in examined farms. Age groups with at least one oral fluid PCV3-positive are marked with a solid green square. An empty green square indicates that oral fluids reacted negative for PCV3. The whisker plot shows minimum and maximum. "X" on axis X indicates age group, which was not sampled. A green mark "X" indicates a group, where oral fluid was not obtained. Age is expressed in weeks.

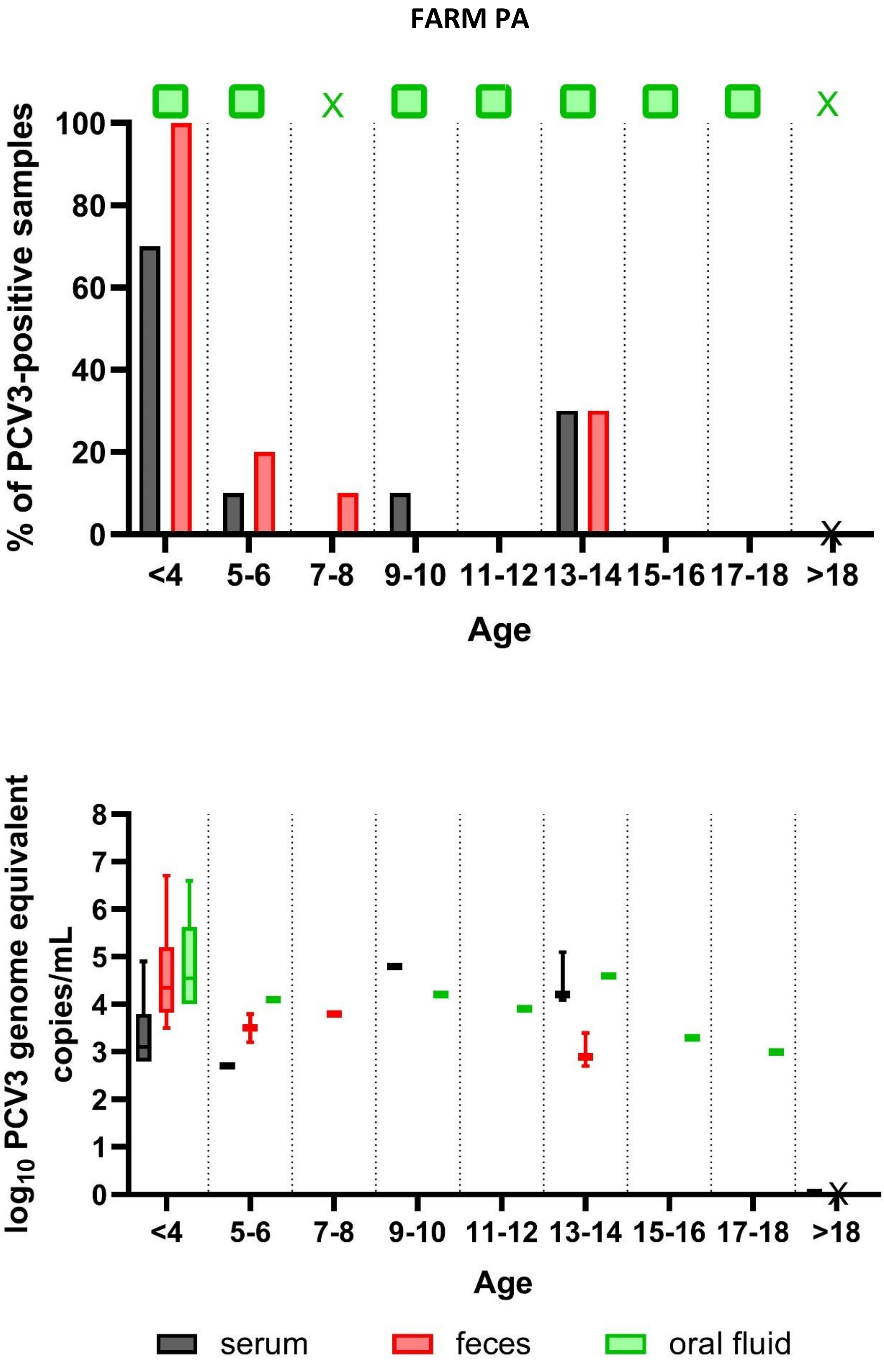

Figure S1: Percentages of porcine circovirus type 3 (PCV3)-positive samples and PCV3 viral loads (log_{10} genome equivalent copies/mL) in different age groups in examined farms. Age groups with at least one oral fluid PCV3-positive are marked with a solid green square. An empty green square indicates that oral fluids reacted negative for PCV3. The whisker plot shows minimum and maximum. "X" on axis X indicates age group, which was not sampled. A green mark "X" indicates a group, where oral fluid was not obtained. Age is expressed in weeks.

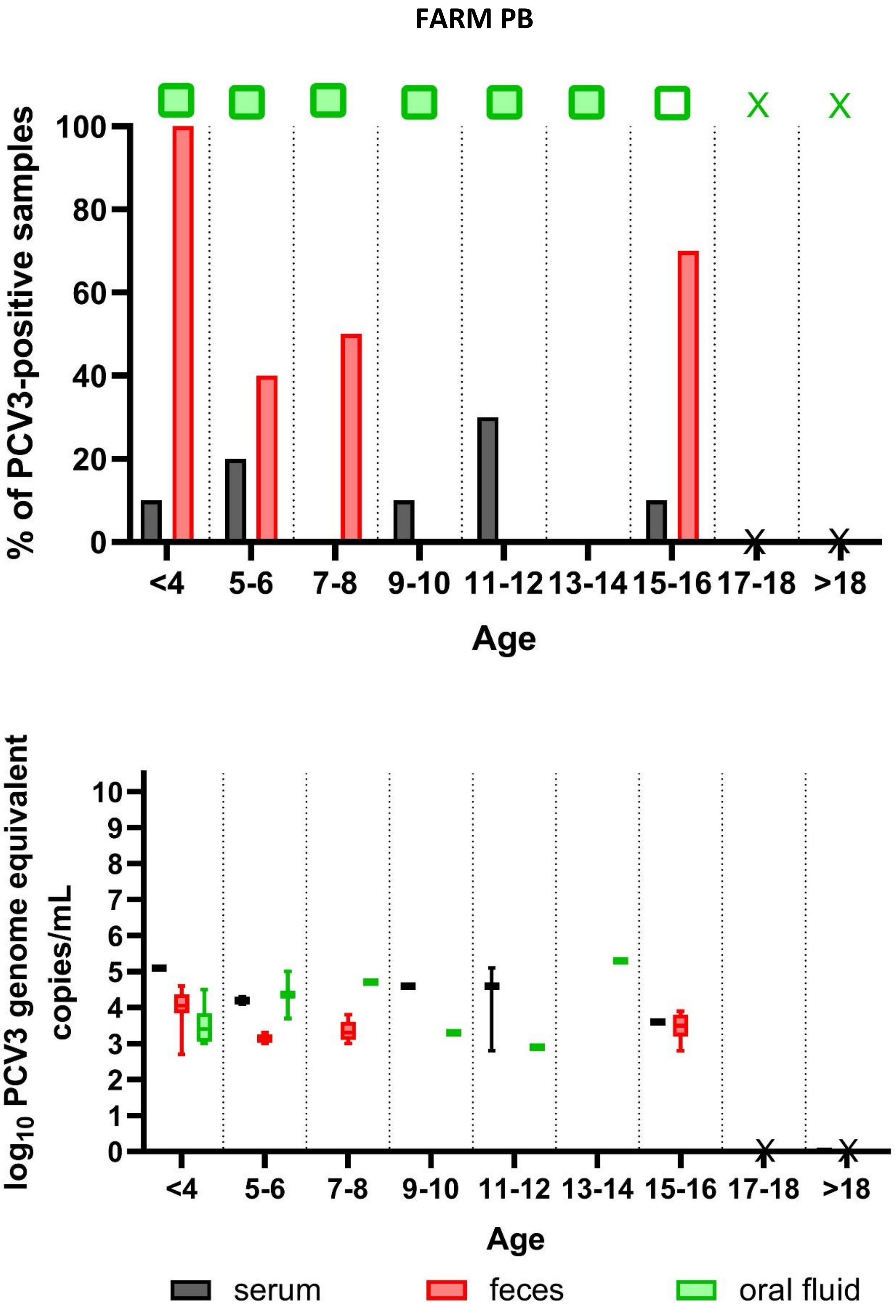

Figure S1: Percentages of porcine circovirus type 3 (PCV3)-positive samples and PCV3 viral loads ($\log_{10}$ genome equivalent copies/mL) in different age groups in examined farms. Age groups with at least one oral fluid PCV3-positive are marked with a solid green square. An empty green square indicates that oral fluids reacted negative for PCV3. The whisker plot shows minimum and maximum. "X" on axis X indicates age group, which was not sampled. A green mark "X" indicates a group, where oral fluid was not obtained. Age is expressed in weeks.

FARM GN

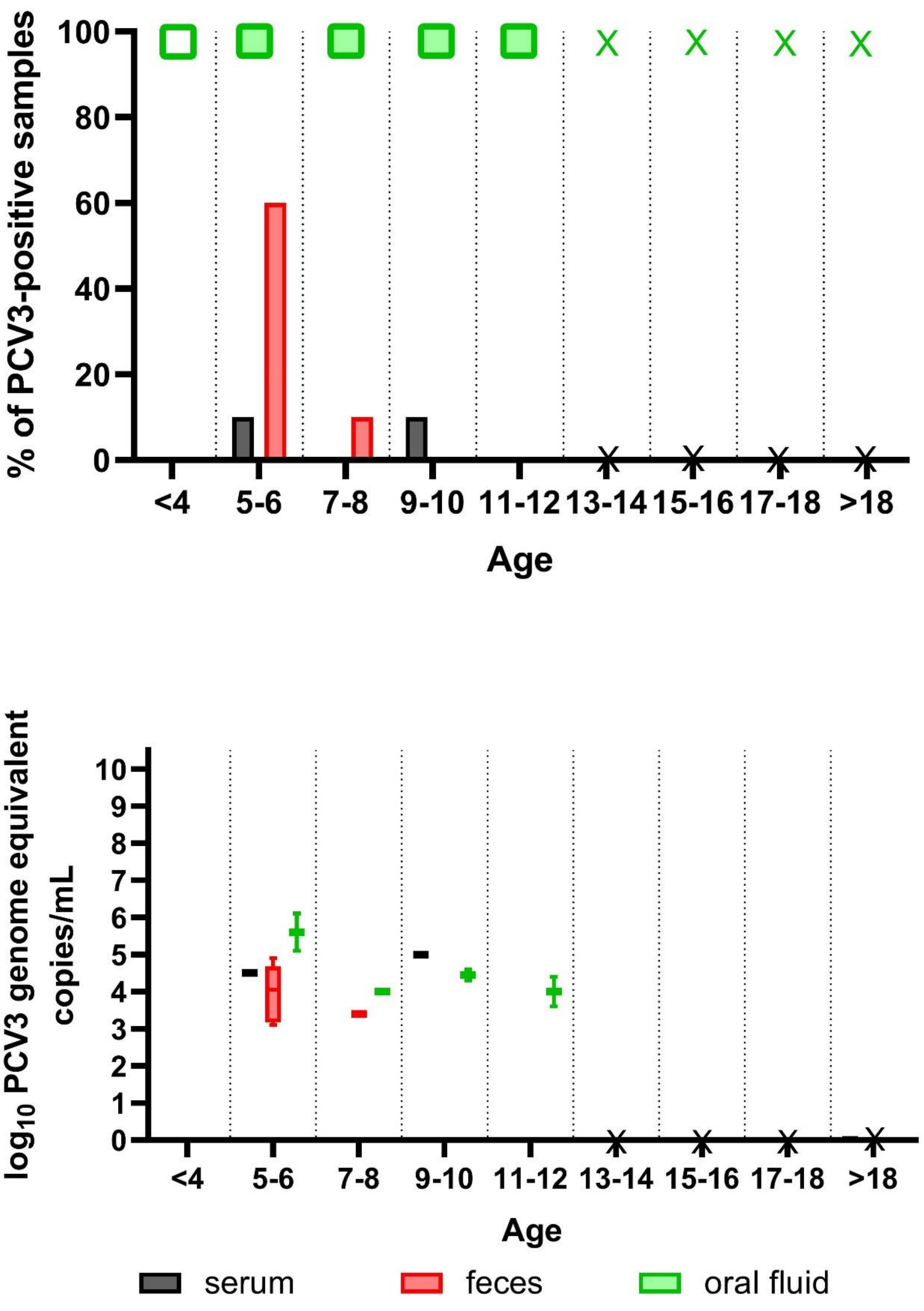

Figure S1: Percentages of porcine circovirus type 3 (PCV3)-positive samples and PCV3 viral loads (log_{10} genome equivalent copies/mL) in different age groups in examined farms. Age groups with at least one oral fluid PCV3-positive are marked with a solid green square. An empty green square indicates that oral fluids reacted negative for PCV3. The whisker plot shows minimum and maximum. "X" on axis X indicates age group, which was not sampled. A green mark "X" indicates a group, where oral fluid was not obtained. Age is expressed in weeks.

FARM DO

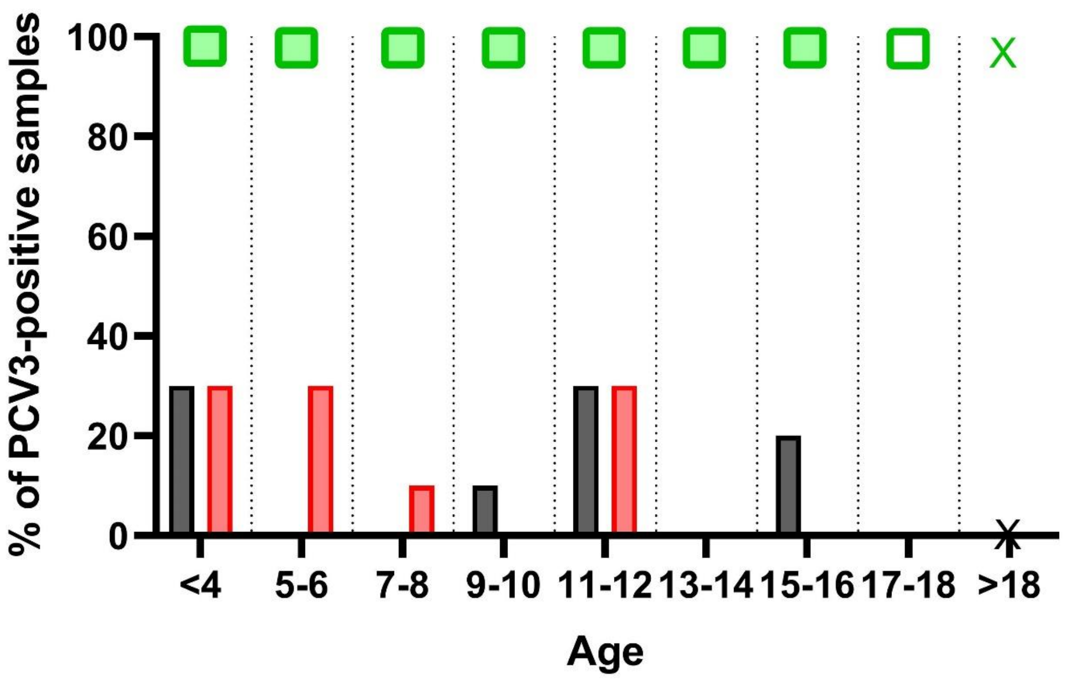

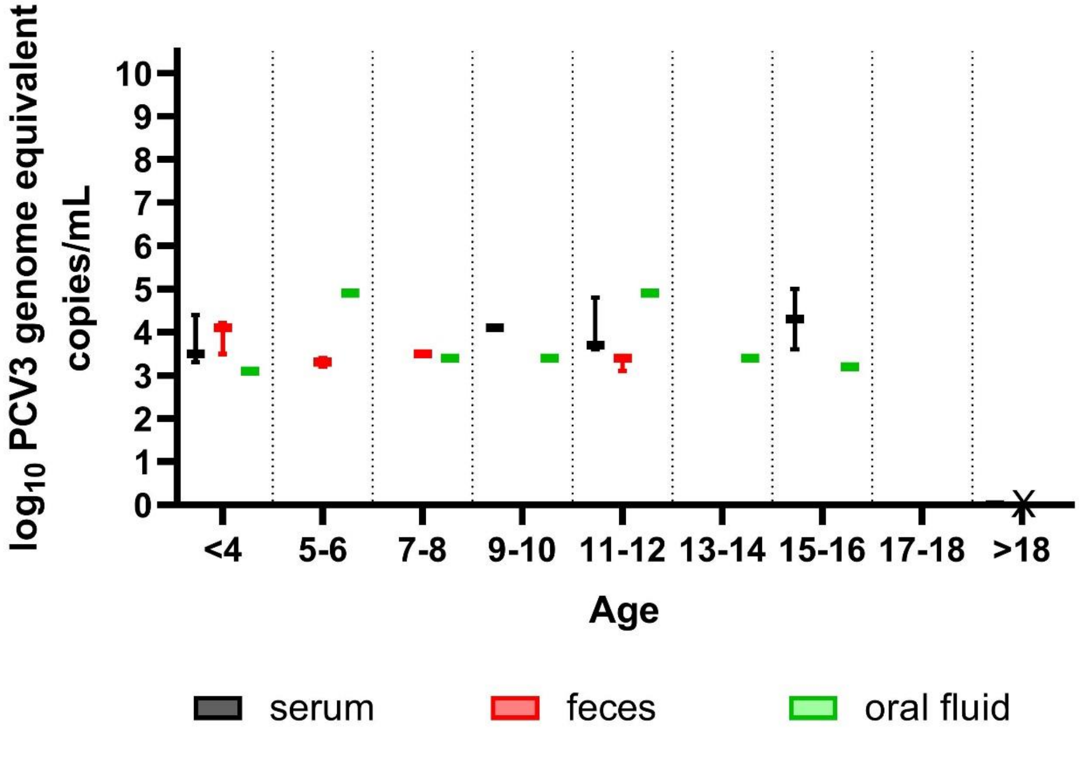

Figure S1: Percentages of porcine circovirus type 3 (PCV3)-positive samples and PCV3 viral loads (log_{10} genome equivalent copies/mL) in different age groups in examined farms. Age groups with at least one oral fluid PCV3-positive are marked with a solid green square. An empty green square indicates that oral fluids reacted negative for PCV3. The whisker plot shows minimum and maximum. "X" on axis X indicates age group, which was not sampled. A green mark "X" indicates a group, where oral fluid was not obtained. Age is expressed in weeks.

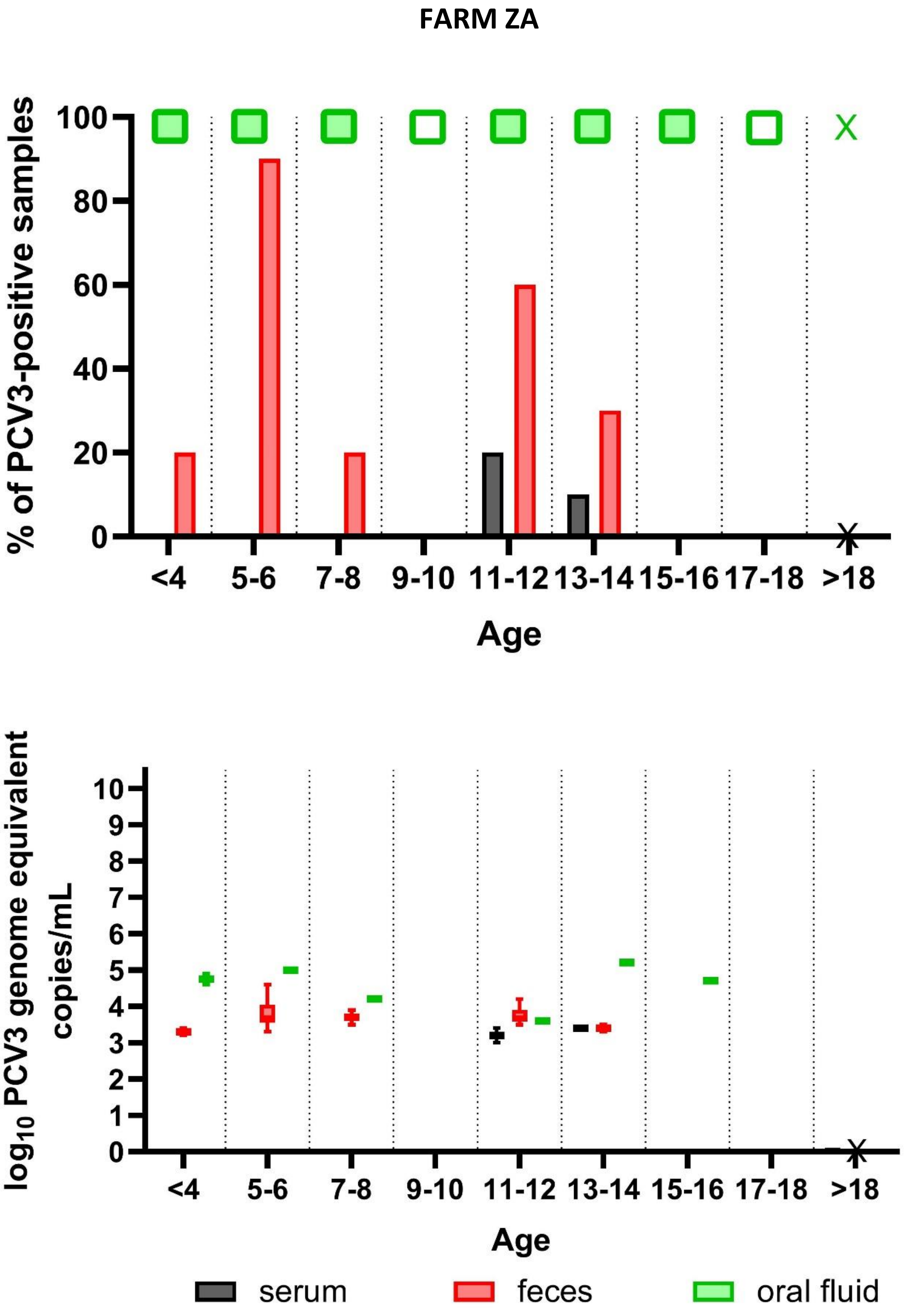

Figure S1: Percentages of porcine circovirus type 3 (PCV3)-positive samples and PCV3 viral loads ($\log_{10}$ genome equivalent copies/mL) in different age groups in examined farms. Age groups with at least one oral fluid PCV3-positive are marked with a solid green square. An empty green square indicates that oral fluids reacted negative for PCV3. The whisker plot shows minimum and maximum. "X" on axis X indicates age group, which was not sampled. A green mark "X" indicates a group, where oral fluid was not obtained. Age is expressed in weeks.

FARM BA

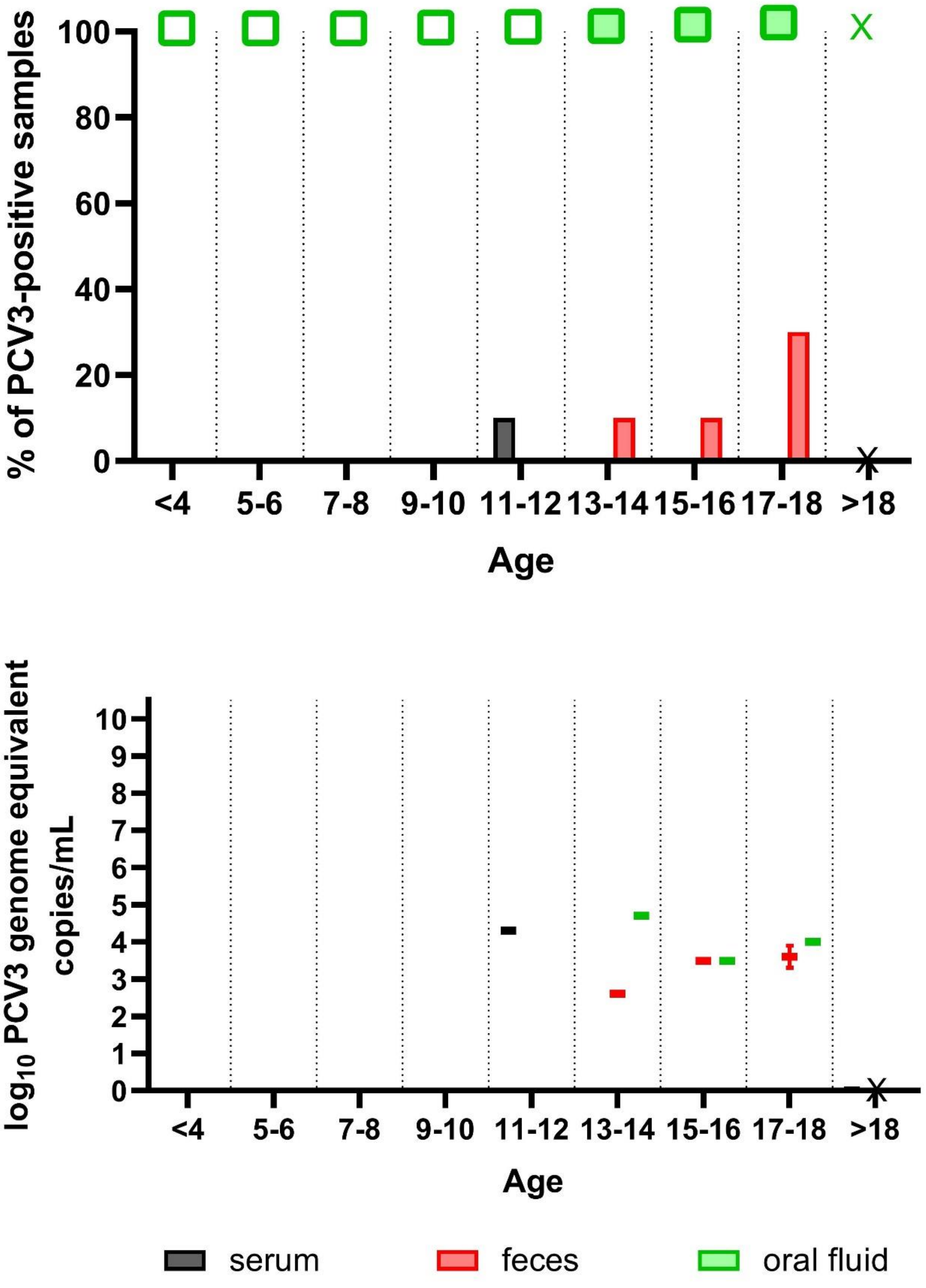

Figure S1: Percentages of porcine circovirus type 3 (PCV3)-positive samples and PCV3 viral loads (log_{10} genome equivalent copies/mL) in different age groups in examined farms. Age groups with at least one oral fluid PCV3-positive are marked with a solid green square. An empty green square indicates that oral fluids reacted negative for PCV3. The whisker plot shows minimum and maximum. "X" on axis X indicates age group, which was not sampled. A green mark "X" indicates a group, where oral fluid was not obtained. Age is expressed in weeks.

FARM B

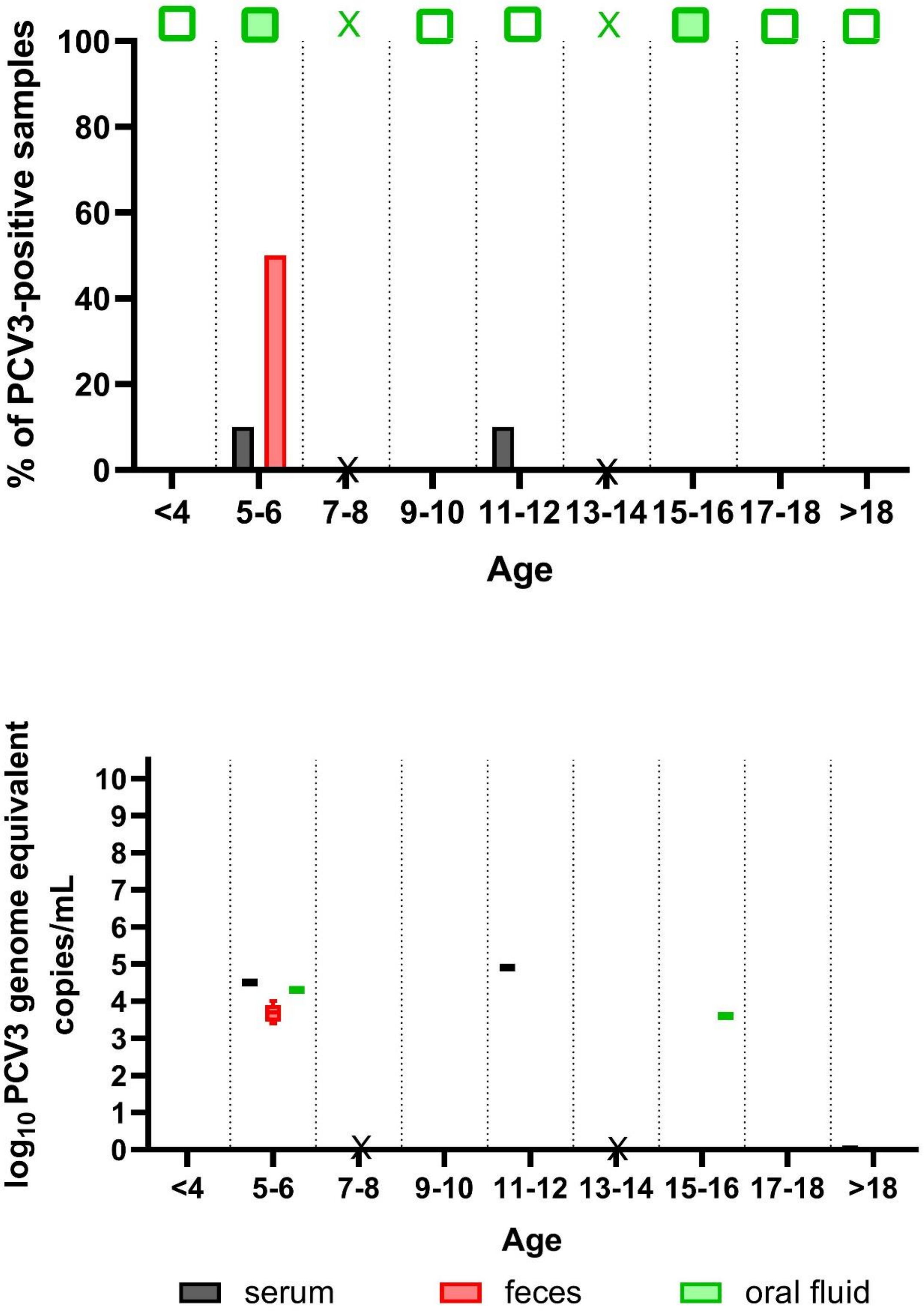

Figure S1: Percentages of porcine circovirus type 3 (PCV3)-positive samples and PCV3 viral loads (log_{10} genome equivalent copies/mL) in different age groups in examined farms. Age groups with at least one oral fluid PCV3-positive are marked with a solid green square. An empty green square indicates that oral fluids reacted negative for PCV3. The whisker plot shows minimum and maximum. "X" on axis X indicates age group, which was not sampled. A green mark "X" indicates a group, where oral fluid was not obtained. Age is expressed in weeks.

FARM PR

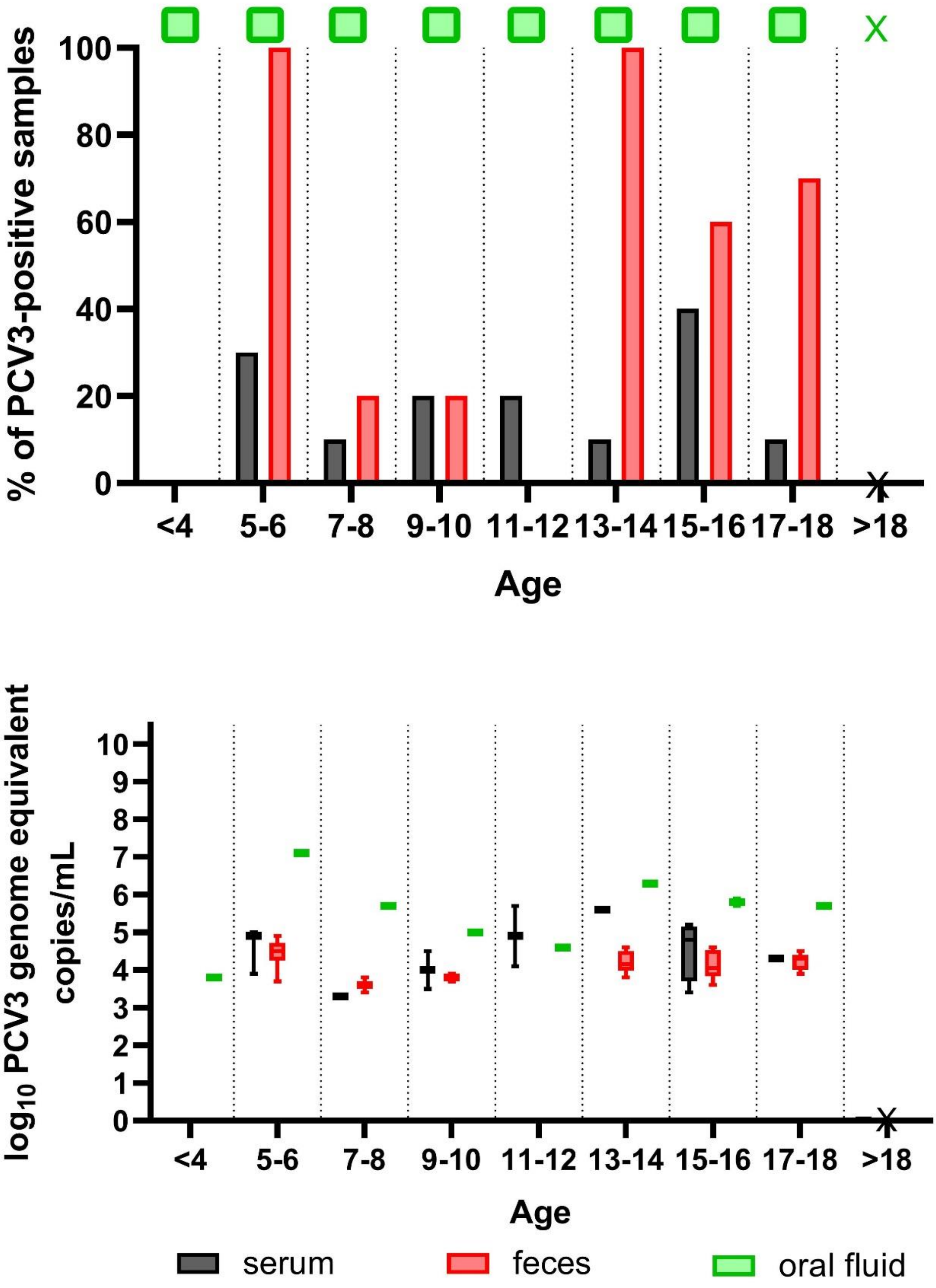

Figure S1: Percentages of porcine circovirus type 3 (PCV3)-positive samples and PCV3 viral loads (log_{10} genome equivalent copies/mL) in different age groups in examined farms. Age groups with at least one oral fluid PCV3-positive are marked with a solid green square. An empty green square indicates that oral fluids reacted negative for PCV3. The whisker plot shows minimum and maximum. "X" on axis X indicates age group, which was not sampled. A green mark "X" indicates a group, where oral fluid was not obtained. Age is expressed in weeks.

FARM WA

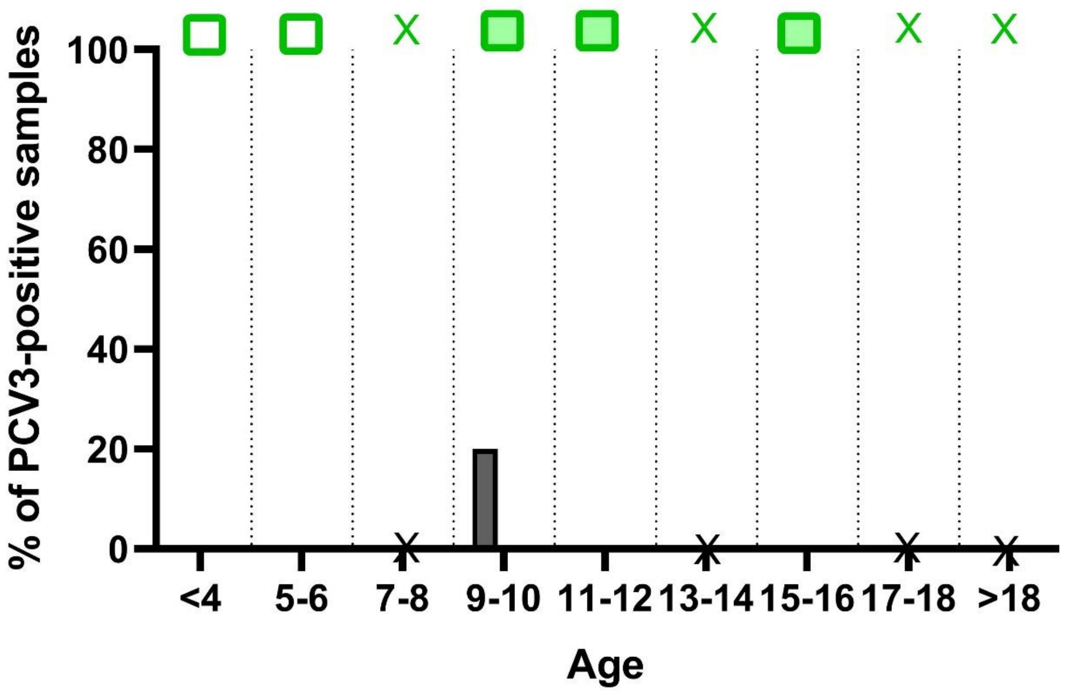

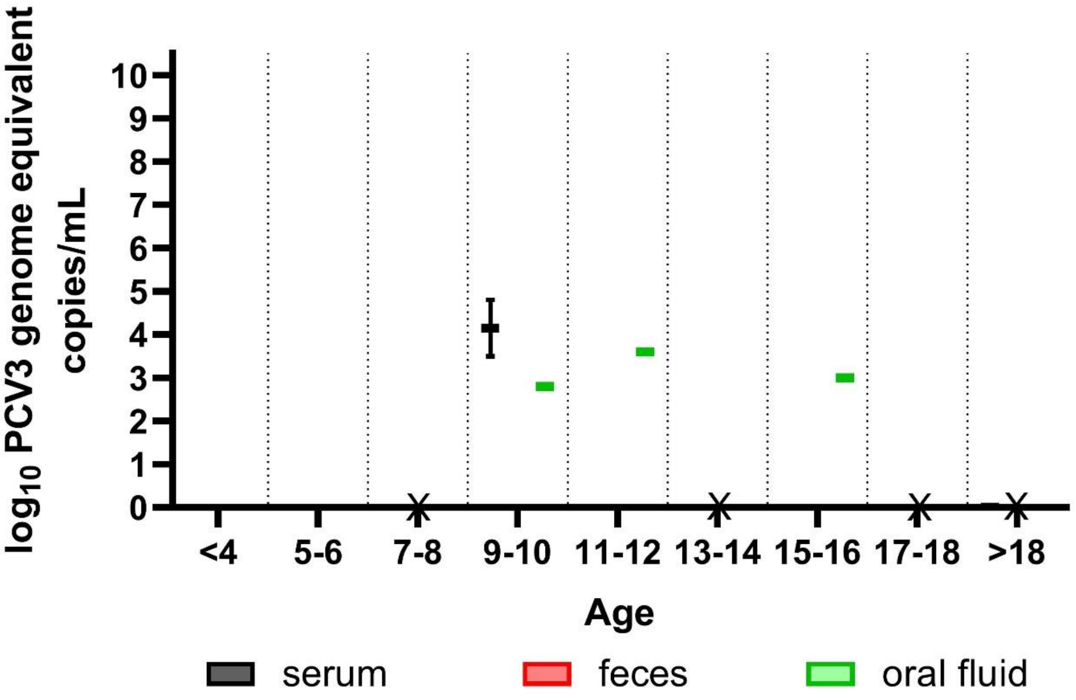

Figure S1: Percentages of porcine circovirus type 3 (PCV3)-positive samples and PCV3 viral loads ($\log_{10}$ genome equivalent copies/mL) in different age groups in examined farms. Age groups with at least one oral fluid PCV3-positive are marked with a solid green square. An empty green square indicates that oral fluids reacted negative for PCV3. The whisker plot shows minimum and maximum. "X" on axis X indicates age group, which was not sampled. A green mark "X" indicates a group, where oral fluid was not obtained. Age is expressed in weeks.

FARM WT

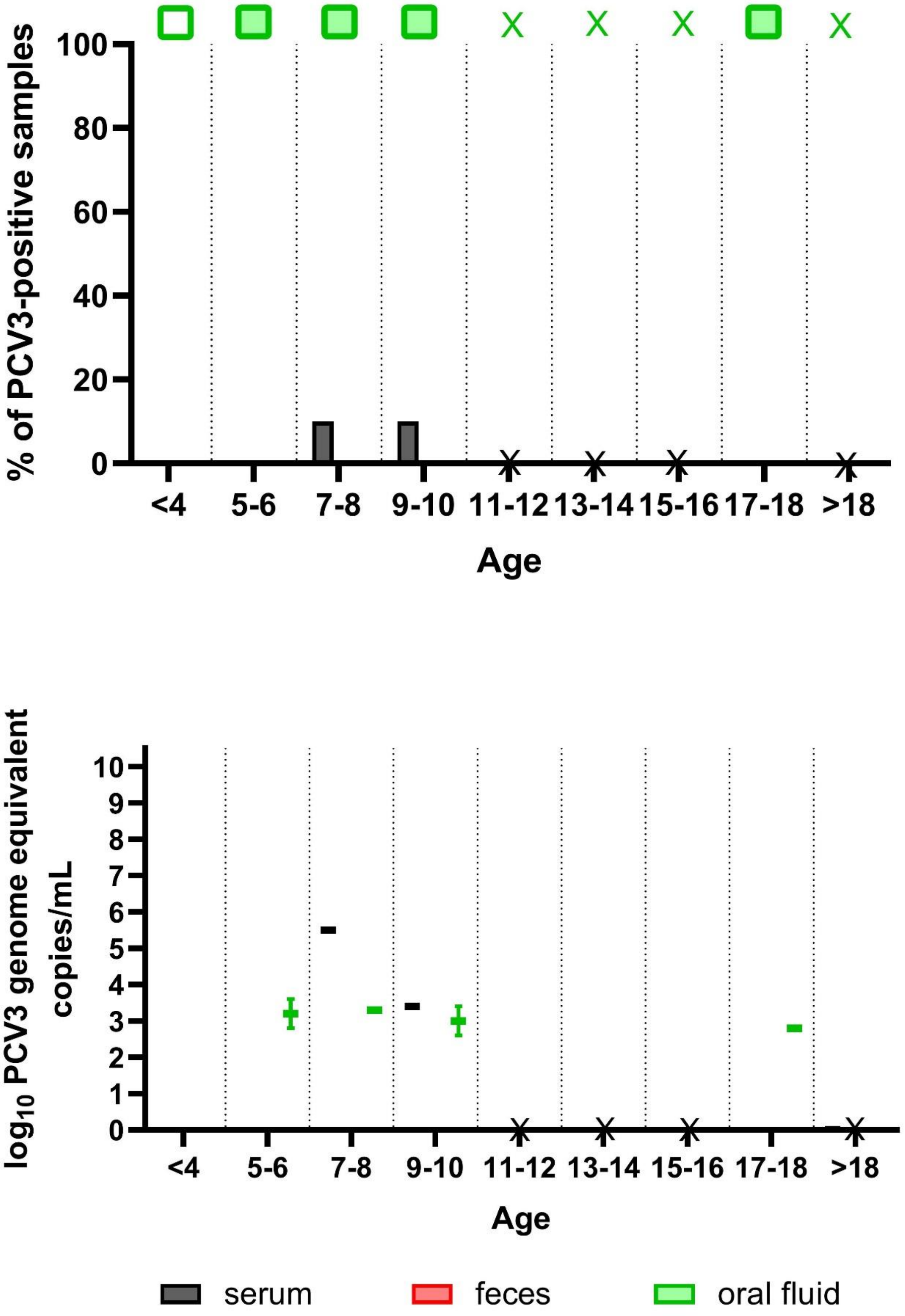

Figure S1: Percentages of porcine circovirus type 3 (PCV3)-positive samples and PCV3 viral loads ($\log_{10}$ genome equivalent copies/mL) in different age groups in examined farms. Age groups with at least one oral fluid PCV3-positive are marked with a solid green square. An empty green square indicates that oral fluids reacted negative for PCV3. The whisker plot shows minimum and maximum. "X" on axis X indicates age group, which was not sampled. A green mark "X" indicates a group, where oral fluid was not obtained. Age is expressed in weeks.

FARM AG

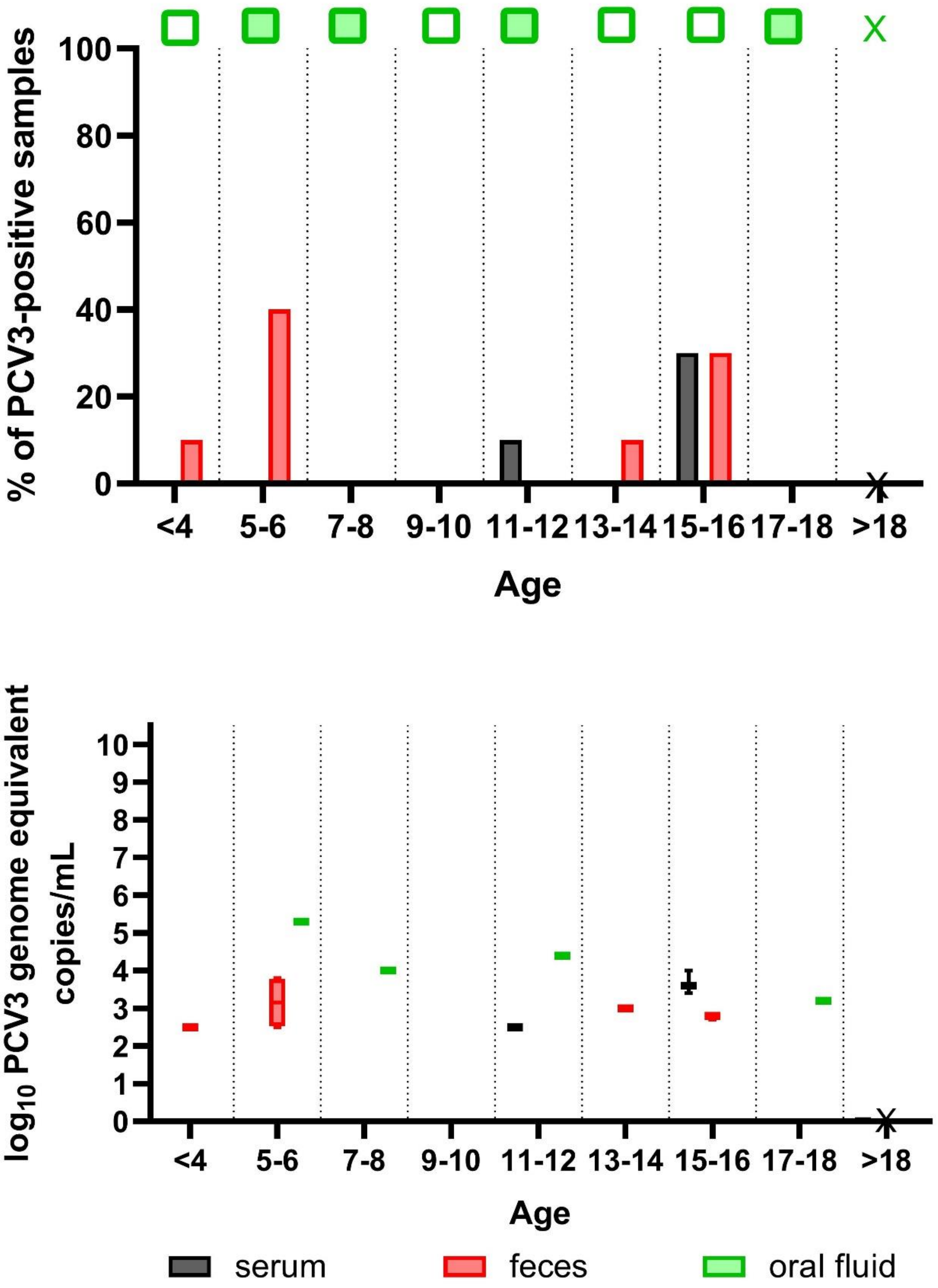

Figure S1: Percentages of porcine circovirus type 3 (PCV3)-positive samples and PCV3 viral loads ($\log_{10}$ genome equivalent copies/mL) in different age groups in examined farms. Age groups with at least one oral fluid PCV3-positive are marked with a solid green square. An empty green square indicates that oral fluids reacted negative for PCV3. The whisker plot shows minimum and maximum. "X" on axis X indicates age group, which was not sampled. A green mark "X" indicates a group, where oral fluid was not obtained. Age is expressed in weeks.

FARM RO

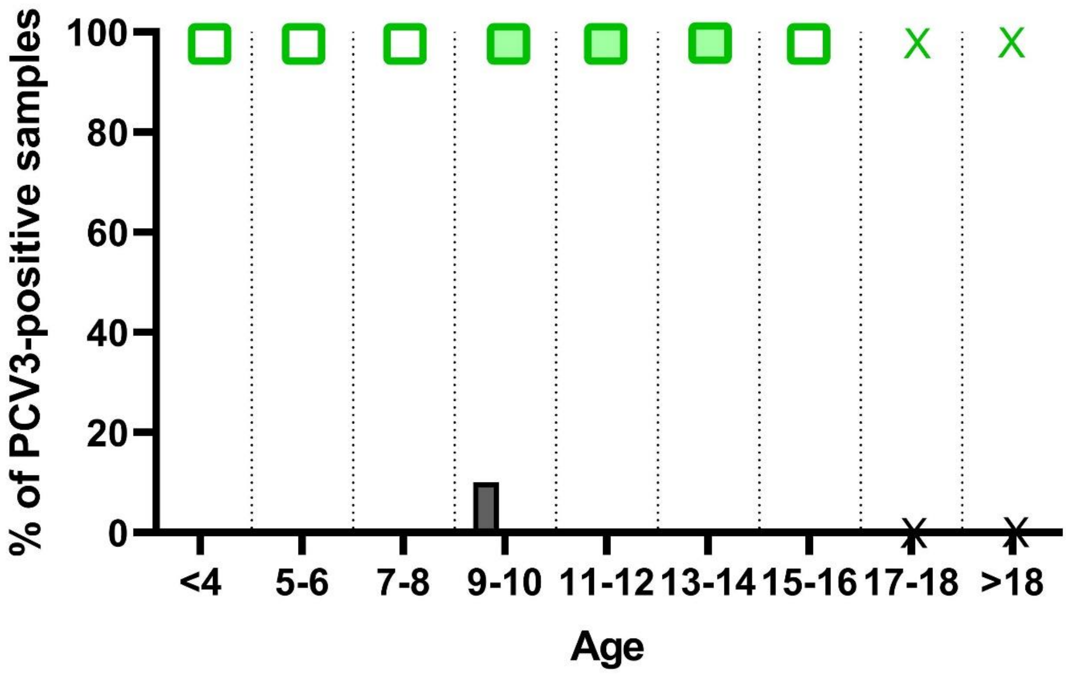

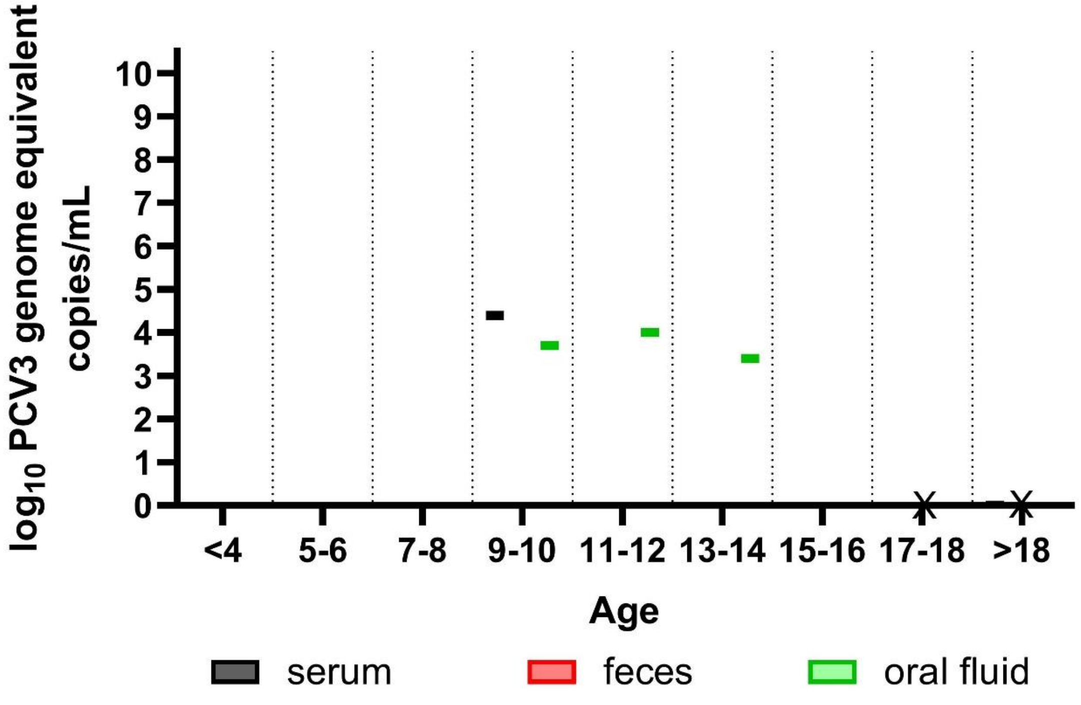

Figure S1: Percentages of porcine circovirus type 3 (PCV3)-positive samples and PCV3 viral loads (log_{10} genome equivalent copies/mL) in different age groups in examined farms. Age groups with at least one oral fluid PCV3-positive are marked with a solid green square. An empty green square indicates that oral fluids reacted negative for PCV3. The whisker plot shows minimum and maximum. "X" on axis X indicates age group, which was not sampled. A green mark "X" indicates a group, where oral fluid was not obtained. Age is expressed in weeks.

FARM GR

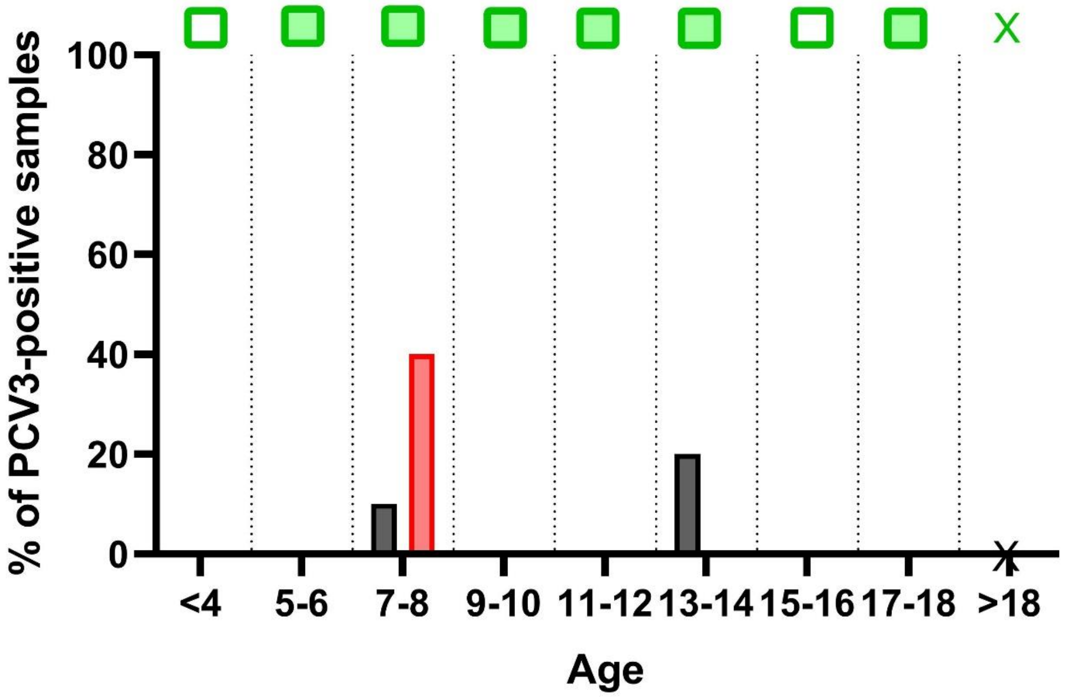

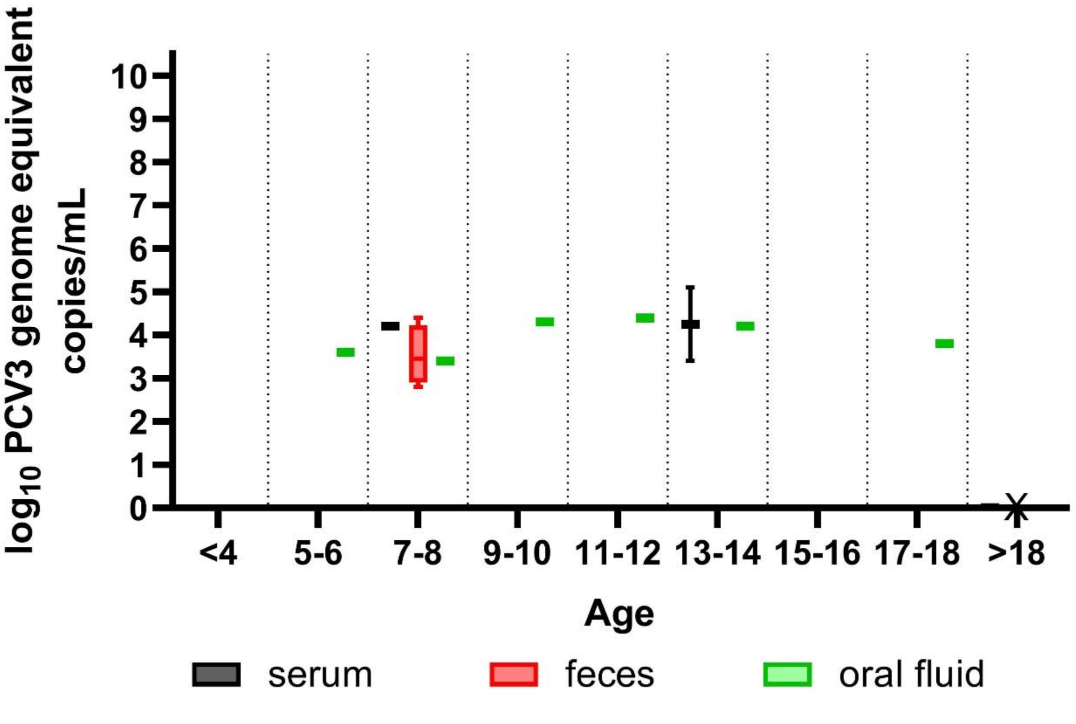

Figure S1: Percentages of porcine circovirus type 3 (PCV3)-positive samples and PCV3 viral loads (log_{10} genome equivalent copies/mL) in different age groups in examined farms. Age groups with at least one oral fluid PCV3-positive are marked with a solid green square. An empty green square indicates that oral fluids reacted negative for PCV3. The whisker plot shows minimum and maximum. "X" on axis X indicates age group, which was not sampled. A green mark "X" indicates a group, where oral fluid was not obtained. Age is expressed in weeks.
