# Supplementary material for: Wide Range of the Prevalence and Viral Loads of Porcine Circovirus Type 3 (PCV3) in Different Clinical Materials from 21 Polish Pig Farms
Source: Pathogens. 2020 May 25;9(5):411. doi: 10.3390/pathogens9050411 (PMC7281387; doi:10.3390/pathogens9050411)
Supplement: Supplementary file 1 [file pathogens-09-00411-s001.zip › supplementary tableS1_rev1_FINAL.pdf]

Farm ID	Stillborn piglet or aborted fetus ID	Sample ID	Fetal samples		PRRSV [3]
			PCV3 viral load [1]	PCV2 viral load [2]	
			log_{10} genome equivalent copies/mL		
**KS**	FM 1	1	-	3.9	-
		2	-	4.6	-
	FM 2	1	-	-	-
		2	-	-	-
	FM 3	1	-	-	-
		2	-	-	-
	FM 4	1	-	-	-
		2	-	-	-
	FM 5	1	-	-	-
		2	-	-	-
	FM 6	1	-	5.5	-
		2	-	-	-
	FM 7	1	-	-	-
		2	-	4.3	-
	FM 8	1	3.6	-	-
		2	-	3.8	-
	FM 9	1	-	5.2	-
		2	-	4.9	-
	FM 10	1	-	-	-
		2	3.8	-	-
	FM 11	1	-	-	-
		2	-	-	-
	FM 12	1	-	-	-
		2	-	-	-
	FM 13	1	3.5	-	-
		2	-	-	-
**PR**	FM 1	1	4.6	-	-
		2	5.0	-	-
	FM 2	1	9.7	4.0	-
		2	10.4	-	-
		3	9.5	-	-
	FM 3	1	6.5	-	-
		2	5.4	-	-
	FM 4	1	6.7	-	-
		2	6.3	-	-
**PA**	FM 1	1	-	-	-
		2	-	-	-
		3	3.6	-	-
	FM 2	1	-	-	-
		2	-	-	-
		3	-	-	-
**ZA**	FM 1	1	-	-	-
		2	-	-	-
		3	-	-	-
		4	-	-	-
	FM 2	1	-	-	-
		2	-	-	-
		3	-	-	-
		4	-	-	-
	FM 3	1	-	-	-
		2	-	-	-
		3	-	-	-
		4	-	-	-
	FM 4	1	-	-	-
		2	-		-
		3	-	-	-
		4	-	-	-
	FM 5	1	-	-	-
	FM 6	1	-	-	-
		2	3.1	-	-
		3	-	-	-

Table S1. Summary results of real-time PCR for porcine circovirus type 3 (PCV3), porcine circovirus type 2 (PCV2) and porcine reproductive and respiratory syndrome virus (PRRSV) in samples collected from stillborn or aborted fetuses (fetal material - FM). "-" indicates result wit Ct>37. From each stillborn piglet or aborted fetus 1-4 pooled samples were prepared consisting of different internal organs (heart, lungs, kidney, liver, spleen), body cavity fluid or umbilical cords.

These samples were tested using real-time PCR assays described in the following articles:

1. Wozniak, A.; Milek, D.; Baska, P.; Stadejek, T. Does porcine circovirus type 3 (PCV3) interfere with porcine circovirus type 2 (PCV2) vaccine efficacy? *Transbound. Emerg. Dis*. **2019**, 66,1454–1461
2. Wozniak, A.; Milek, D.; Matyba, P.; Stadejek, T. Real-time PCR detection patterns of porcine circovirus type 2 (PCV2) in Polish Farms with different Status of vaccination against PCV2. *Viruses* **2019**, 11(12), 1135
3. Stadejek, T.; Larsen, L.E.; Podgorska, K.; Botner, A.; Botti, S.; Dolka, I.; Fabisiak, M.; Heegaard, P.M.H.; Hjulsager, Ch.K.; Huc, T.; Kvisgaard, L.K.; Sapierzynski, R.; Nielsen, J. Pathogenicity of three genetically diverse strains of PRRSV Type 1 in specific pathogen free pigs. *Vet. Microbiol.* **2017**, 209, 13-19
